# ALK inhibitors for non-small cell lung cancer: A systematic review and network meta-analysis

**DOI:** 10.1371/journal.pone.0229179

**Published:** 2020-02-19

**Authors:** Jesse Elliott, Zemin Bai, Shu-Ching Hsieh, Shannon E. Kelly, Li Chen, Becky Skidmore, Said Yousef, Carine Zheng, David J. Stewart, George A. Wells

**Affiliations:** 1 Cardiovascular Research Methods Centre, University of Ottawa Heart Institute, Ottawa, Canada; 2 Independent Information Specialist, Ottawa, Canada; 3 Division of Medical Oncology, University of Ottawa and The Ottawa Hospital, Ottawa, Canada; Laurentian University, CANADA

## Abstract

**Background:**

We sought to assess the relative effects of individual anaplastic lymphoma kinase (ALK) inhibitors for the treatment of non-small cell lung cancer (NSCLC).

**Methods:**

We searched MEDLINE, Embase, Cochrane CENTRAL, and grey literature (July 23, 2019) for randomized controlled trials (RCTs) that included participants with *ALK-* or *ROS1*-positive NSCLC who received any ALK inhibitor compared with placebo, another ALK inhibitor, or the same ALK inhibitor at a different dose. The primary outcome was treatment-related death. Secondary outcomes were overall survival (OS), progression-free survival (PFS), and serious adverse events. Data were pooled via meta-analysis and network meta-analysis, and risk of bias was assessed. PROSPERO: CRD42017077046.

**Results:**

Thirteen RCTs reporting outcomes of interest among participants with *ALK-*positive NSCLC were identified. Treatment-related deaths were rare, with 10 deaths attributed to crizotinib (risk difference v. chemotherapy: 0.49, 95% credible interval [CrI] –0.16 to 1.46; odds ratio 2.58 (0.76–11.37). All ALK inhibitors improved PSF relative to chemotherapy (hazard ratio [95% CrI]: crizotinib 0.46 [0.39–0.54]; ceritinib 0.52 [0.42–0.64]; alectinib 300 BID 0.16 [0.08–0.33]; alectinib 600 BID 0.23 [0.17–0.30]; brigatinib 0.23 [0.15–0.35]), while alectinib and brigatinib improved PFS over crizotinib and ceritinib (alectinib v. crizotinib 0.34 [0.17–0.70]; alectinib v. ceritinib 0.30 [0.14–0.64]; brigatinib v. crizotinib 0.49 [0.33–0.73]; brigatinib v. ceritinib 0.43 [0.27–0.70]). OS was improved with alectinib compared with chemotherapy (HR 0.57 [95% CrI 0.39–0.83]) and crizotinib (0.68 [0.48–0.96]). Use of crizotinib (odds ratio 2.08 [95% CrI 1.56–2.79]) and alectinib (1.60 [1.00–2.58]) but not ceritinib (1.25 [0.90–1.74), increased the risk of serious adverse events compared with chemotherapy. Results were generally consistent among treatment-experienced or naïve participants.

**Conclusion(s):**

Treatment-related deaths were infrequent among *ALK*-positive NSCLC. PFS may be improved by alectinib and brigatinib relative to other ALK inhibitors; however, the assessment of OS is likely confounded by treatment crossover and should be interpreted with caution.

## Background

Lung cancer ranks among the most common types of cancer in North America, with an estimated incidence of 222,500 in the US and 28,600 in Canada in 2017.[1, 2] About 85% of patients with lung cancer have non-small cell lung cancer (NSCLC), with 5% and 1% of patients harbouring rearrangements in the anaplastic lymphoma kinase (*ALK*) gene or *ROS1* gene, respectively.[3] The presence of mutations or rearrangements in the *ALK* gene renders the cancer sensitive to tyrosine kinase inhibitors, which bind to receptor tyrosine kinases and inhibit downstream signalling pathways.[4]

Four ALK inhibitors are approved for use in Canada and the US: crizotinib, ceritinib, and alectinib, and brigatinib. Crizotinib, the first-in-class tyrosine kinase inhibitor, was initially approved for use in *ALK*-positive NSCLC patients in the US in 2011 and in Canada in 2012 based on initial results from single-arm phase I and II trials (PROFILE 1001, PROFILE 1005), which reported median progression-free survival of 8–10 months among participants with previous treatment experience. Subsequent randomized controlled trials (RCTs) comparing crizotinib with chemotherapy in treatment-experienced (PROFILE 1007) or naïve (PROFILE 1014) patients reported significant improvements in progression-free survival but no corresponding improvement in overall survival; however, a high proportion of patients who experienced disease progression on chemotherapy crossed over to crizotinib treatment, confounding the results. Acquired resistance to crizotinib is common, via secondary mutations in the *ALK* gene, which limits its efficacy to a median of about one year.[4] Second-generation ALK tyrosine kinase inhibitors (ceritinib, alectinib, brigatinib) were developed to overcome crizotinib resistance,[4] although they may also be effective as first-line treatments. Among treatment-naïve patients, alectinib may improve progression-free survival with fewer serious adverse events (SAEs) compared with crizotinib,[5] although resistance to second-generation ALK inhibitors has also been reported.[4] Additional treatment options include the third-generation ALK inhibitors lorlatinib, entrectinib, and ensartinib.[4]

Previous systematic reviews of ALK inhibitors for the treatment of NSCLC have reported improved overall and progression-free survival with crizotinib and alectinib compared with chemotherapy.[6, 7] However, previous reviews were limited by the use of a pair-wise meta-analysis approach, which permits comparison of only two therapies at one time (e.g., alectinib v. chemotherapy). When choosing between treatment options, clinicians require information about the relative effectiveness and safety of all available options. As an extension of traditional pair-wise meta-analysis, network meta-analysis (NMA) allows the comparison of multiple treatments at one time and provides estimates of their relative effectiveness and safety, which is more informative for clinical decision-making. In this study, we performed a comprehensive systematic review to identify all RCTs involving the use of any ALK inhibitor to treat *ROS1* or *ALK*-positive NSCLC, and we used NMA methodology to provide an estimate of progression-free survival, overall survival, and SAEs associated with each individual ALK inhibitor.

## Methods

This review was registered *a priori* (PROSPERO no.: CRD42017077046) and followed the Cochrane Handbook for Systematic reviews for Interventions[8] and the PRISMA for Network Meta-Analysis checklist[9] (S1 File Appendix 1).

### Search strategy

Using the OVID platform, we searched Embase, Ovid MEDLINE, MEDLINE In-Process & Other Non-Indexed Citations, as well as the Cochrane Library on Wiley (July 23, 2019). The search strategy was peer-reviewed by use of the PRESS checklist[10] and utilized a combination of controlled vocabulary (e.g., “Carcinoma, Non-Small-Cell Lung”) and keywords (e.g., “NSCLC”, “ALK inhibitors”), with vocabulary and syntax adjusted across databases (S1 File Appendix 2). We also performed a targeted search of the grey literature, including searching ClinicalTrial.gov, ICTRP Search Portal, and the websites of major government regulatory agencies. There were no date or language limits.

### Study selection

Titles and abstracts of identified records, and the full-text of any potentially relevant record, were evaluated by two independent reviewers; disagreements were resolved by consensus. Decisions about study eligibility were based on information provided in the published records; study authors were not contacted to clarify eligibility.

### Eligibility

The following eligibility criteria were applied to each identified record to determine eligibility:

#### Population

Treatment-naïve or experienced participants with phase III or IV *ALK*-positive and/or *ROS1*-positive NSLC.

#### Interventions

ALK inhibitors (e.g, crizotinib, ceritinib, alectinib, brigatinib, loratinib, ensartinib, and entrectinib).

#### Comparators

Placebo, chemotherapy, radiotherapy, another ALK inhibitor, or the same ALK inhibitor at a different dose.

#### Outcomes

The primary outcome was treatment-related death. Secondary outcomes were overall survival, progression-free survival, and SAEs as reported by the study authors. Studies were not selected for inclusion based on reported outcomes.

#### Study design

Randomized controlled trials.

### Data extraction and risk of bias

Data were extracted by one reviewer and verified for completeness and accuracy by a second reviewer, with disagreements resolved by discussion. We extracted study characteristics (e.g., author, year of publication) and participant characteristics (e.g., age, sex, treatment history, comorbidities), as well as outcome data. We extracted event counts and denominators (number analyzed) for dichotomous outcomes (treatment-related deaths, SAEs), and we extracted hazard ratios (HRs) and 95% confidence intervals for time-to-event outcomes (overall survival, progression-free survival). Where PFS were assessed by both study investigators as well as by independent review committees, we extracted and analyzed data from the latter. We also extracted the percentage of participants who remained alive (overall survival) or free of disease progression (progression-free survival) after 12 months of treatment; these data are narratively described. We compared study and patient characteristics across studies to ensure that each record represented a unique publication of study data and to match up companion publications (i.e., multiple records pertaining to a single RCT). Risk of bias (ROB) was assessed by two independent reviewers by use of the Cochrane Collaboration’s ROB tool for RCTs.[11] Specifically, we assigned a judgment of high, low, or unclear ROB for the domains allocation concealment, randomization, blinding, incomplete outcome data, and selective outcome reporting, as well as other biases stemming from issues such as early study termination. Risk of bias related to blinding was assessed separately for personnel/participants, subjective outcomes, and objective outcomes.

### Data analysis

We first performed pair-wise meta-analysis to explore the class effect of treatment with any ALK inhibitor versus chemotherapy, followed by NMA to explore the effect of individual ALK inhibitors. Base-case analyses involving all participants were performed for all outcomes (treatment-related death, overall survival, progression-free survival, SAEs) as were subgroup analyses based on treatment experience. Complete case analyses were performed for dichotomous outcomes (treatment-related deaths, SAEs). For all other outcomes, analyses involved HRs reported by study authors, which accounted for participants censored from the study. Analyses were stratified by treatment experience (naive or experienced) for all meta-analyses and NMAs; no additional subgroup analyses were performed. Bayesian meta-analyses and NMA were performed by use of WinBUGS (v.1.4.3; MRC Biostatistics Unit). Chemotherapy was selected as the reference group for the MA and NMA comparisons. We assessed heterogeneity by use of the *I*^2^ value, with *I*^2^ values above 75% considered to represent high heterogeneity; data were not pooled if the *I*^2^ value exceeded this threshold. We also considered clinical heterogeneity across RCTs by evaluating the similarity of included participants. Additionally, we assessed the model fit (fixed versus random effects) based on the deviance information criterion (DIC) and by comparing the residual deviance to the number of unconstrained data points for each analysis.[12]

In the Bayesian MA and NMAs, a normal likelihood with identity link model was applied for the time-to-event outcomes (overall survival, progression-free survival) using study-level summary measures (log HRs and their standard errors). A binomial likelihood model with logit link was used for the Bayesian MA and NMAs for the dichotomous outcomes (treatment-related death, SAEs) to estimate relative risk (RR) and risk difference (RD). Point estimates (odds ratios [ORs], RR, RD for dichotomous outcomes, HRs for time-to-event outcome) and 95% credible intervals (CrIs) were estimated using Markov Chain Monte Carlo methods. For dichotomous outcomes, the RR was estimated based on the OR and the mean proportion of patients who experience the outcome in the reference group of the included studies. The conversion of OR to RR was based on the incidence of the event in the reference group. Vague priors (N (0, 100^2^)) were assigned for basic parameters of the treatment effect in the model. Informative priors (Log normal (-3.02, 1.85^2^)) were applied for the between-study variance parameter in the random-effect binomial likelihood model for dichotomous outcomes to improve precision and reduce heterogeneity between studies.[13] Model convergence was assessed by use of model diagnostics (trace plots, Brooks–Gelman–Rubin statistic).[14] Three chains were fit into WinBUGS for each analysis, each employing ≥ 10,000 iterations, with a burn-in of ≥ 10,000 iterations. Inconsistency between direct evidence and indirect evidence was formally assessed using the posterior mean deviance of the individual data points in the inconsistency model plotted against their posterior mean deviance in the consistency model if there were closed loops in the networks[15] For networks without a closed loop, we assessed exchangeability by comparing the study and patient characteristics to ensure that they satisfied the assumption that all patients were equally likely to receive a given treatment in the network. All network diagrams were constructed using NodeXL (Social Media Research Foundation).

## Results

### Search results and study characteristics

**Fig 1:**
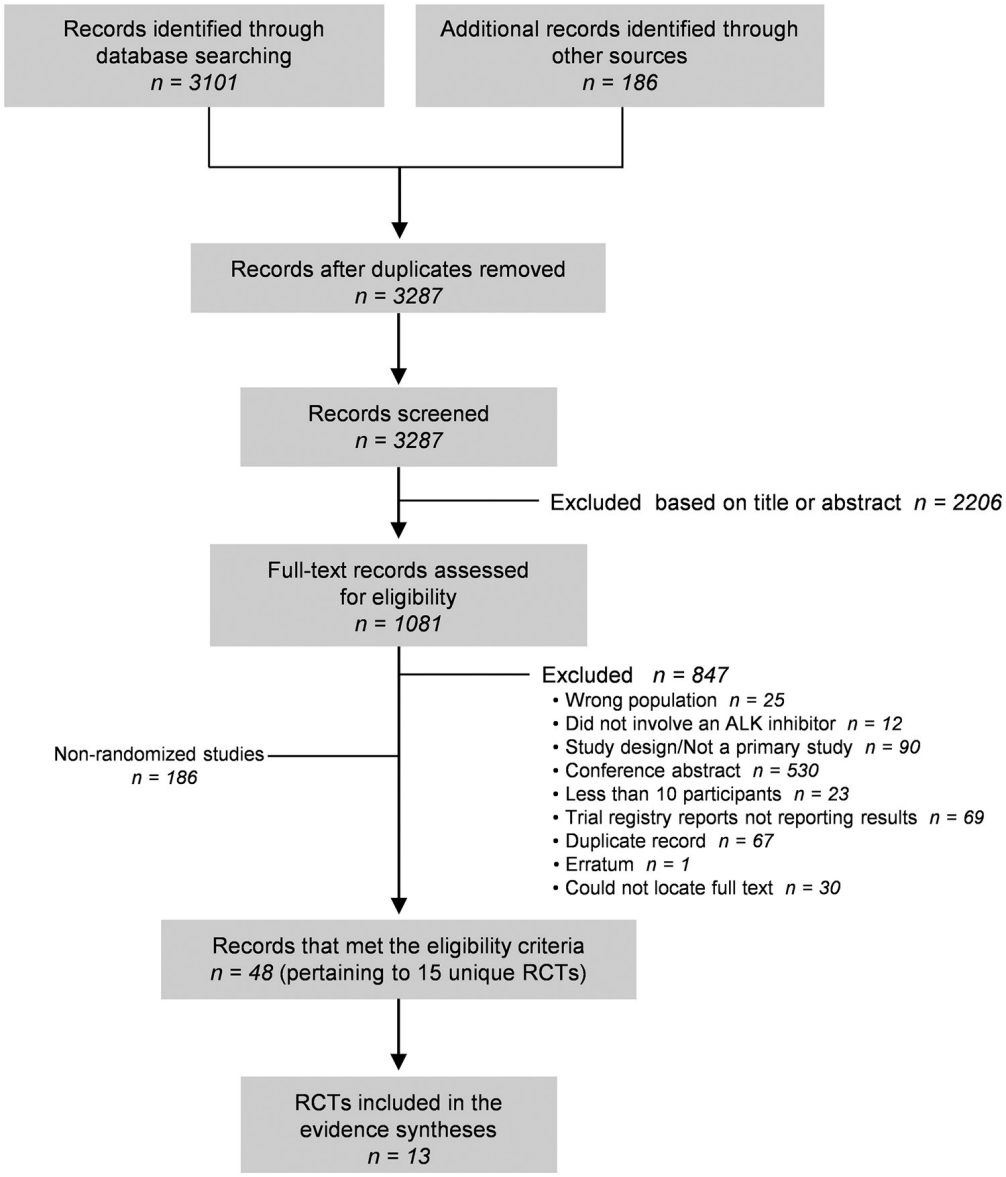
PRISMA flow diagram. RCT = randomized controlled trial, NRS = non-randomized study.

In total, 3287 records were identified from the literature search (Fig 1). After full-text evaluation of 1081 records, we included 48 records pertaining to 15 unique RCTs.[5, 16–29] The complete list of included records is shown in S1 File Appendix 3). Of these, 13 RCTs[5, 16–19, 21–26, 28, 29] reported on outcomes of interest for this review, predominantly involving two-arm parallel-group designs (12 RCTs), with one crossover design[19] (Table 1). All were published after 2013, with between 28 and 376 participants with *ALK*-positive NSCLC. No RCTs involved participants with *ROS1* NSCLC. Seven RCTs[16–18, 23, 24, 26, 28] compared an ALK inhibitor to chemotherapy, while six RCTs[5, 19, 21, 22, 25, 29] involved head-to-head comparison of one ALK inhibitor to another ALK inhibitor or to the same inhibitor at a different dose. In total, eight RCTs involved crizotinib,[5, 16–18, 21, 25, 28, 29] five involved alectinib,[5, 19, 21, 26, 29] two involved ceritinib,[23, 24] and two involved brigatinib.[22, 25] Among the parallel-group RCTs, participants were allowed to cross to the alternative treatment group after disease progression in six trials.[17, 22–26]

**Table 1:** Study characteristics of included randomized controlled trials.

Author, yr, page (study name; NCT no.) (companion publications)	Population	Groups (no. randomized)	Duration of treatment, median (IQR), months	Cross-over between treatment groups allowed?	Reported outcomes of interest to this review	Funding source
**Chemotherapy-controlled**						
Wu 2018, p. 1549 (PROFILE 1029; NCT01639001)[28, 30]	18–70 years, ALK-positive NSCLC, with ECOG score of 0–2, with no prior systemic treatment	Crizotinib 250 mg BID (104) Chemotherapy (103)	NR	Not reported	TR death; OS; PFS (independent review)*;	Pharma
Shaw 2013, p. 2385 (PROFILE 1007; NCT00932893)[16, 31] (Blackhall 2014[32])	≥ 18 yr, ALK-positive NSCLC, with ECOG score of 0–2, with progressive disease after one prior platinum-based chemotherapy regimen	Crizotinib, 250 mg BID (173) Chemotherapy (174)	NR	Not during study period; participants from the chemotherapy arm could enroll in NCT00932451	TR death; OS; PFS (independent radiologic review*)	Pharma
Solomon 2014, p. 2167[25] (PROFILE 1014; NCT01154140) (Thorne-Nuzzo 2017,[33] Solomon 2016[34], Solomon 2018[35])	≥ 18 yr, ALK-positive NSCLC, with ECOG score of 0–2, with no prior systemic treatment	Crizotinib 250 mg BID (172) Chemotherapy (171)	10.9 (range 0.4 to 34.3) 4.1 (range 0.7 to 6.2)	Yes; participants in the chemotherapy arm with disease progression could cross to the crizotinib arm provided safety criteria were met	TR death; OS; PFS (independent review)*	Pharma
Zhao 2015, p. 616[18]	≥ 18 yr, ALK-positive NSCLC, Karnofsky performance status (KPS) score ≥ 70, following first- or second-line chemotherapy	Crizotinib, 250 mg BID (14) Chemotherapy (14)	NR	Not reported	TR death; SAEs	Non-pharma
Novello 2018, p. 1409 (ALUR; NCT02604342) [26]	ALK-positive NSCLC, with ECOG score of 0–2; two prior lines of systemic therapy including one line of chemotherapy and one of crizotinib	Alectinib 600 mg BID (72) Chemotherapy (35)	20.1 wk (range 0.4–62.1) 6.0 wk (range 1.9–47.1)	Yes; cross-over from chemotherapy to alectinib was permitted following progression	OS; PFS (investigator-assessed)*	Pharma
Soria 2017, p. 917[24, 36] (ASCEND-4; NCT01828099)	≥ 18 yr, ALK-positive NSCLC, ECOG score of 0–2, previously untreated	Ceritinib 750 mg QD (189) Chemotherapy (187)	66.4 (30.8 to 83.7) 29.9 (13.0 to 62.3)	Yes, participants in the chemotherapy arm could crossover to ceritinib after disease progression	TR death; OS; PFS (independent review)*; SAEs	Pharma
Shaw 2017, p. 874 (ASCEND-5, NCT01828112)[23, 37] (Kiura 2018[38])	≥ 18 yr, ALK-positive NSCLC, with WHO performance status of 0–2, one or two previous chemotherapy regimens and previous crizotinib for at least 21 d	Ceritinib 750 mg QD (115) Chemotherapy (116)	30.3 (13.3 to 54.1) 6.3 (6.0 to 15.1)	Yes, participants in the chemotherapy arm could cross over to the ceritinib group after disease progression	TR death; OS; PFS (independent review)*	Pharma
**Head-to-head comparisons of ALK inhibitors**						
Zhou 2019, p. 437 (ALESIA; NCT02838420)[29]	≥ 18 yr, ALK-positive NSCLC, ECOG score of 0–2, life expectancy of >12wk, no prior systemic therapy	Crizotinib 250 mg BID (62) Alectinib 600 mg BID (125)	12.6 14.7	No	TR death; OS; PFS (investigator assessed)*; SAEs	Pharma
Camidge 2018, p. 1 (ALTA-1L; NCT02737501)[25]	≥ 18 yr, ALK-positive locally advanced or metastatic NSCLC, with at least one measurable lesion, and no prior ALK-targeted therapy	Crizotinib 250 mg BID (138) Brigatinib 180 mg QD (137)	7.4 (range 0.1 to 19.2) 9.2 (range 0.1 to 18.4)	Yes: patients in the crizotinib group could cross over to brigatinib after disease progression	TR death; OS; PFS (independent review)*	Pharma
Peters 2017, p. 829 (ALEX; NCT02075840)[5, 39] (Camidge 2019[40]; Gadgeel 2018[41])	≥ 18 yr, ALK-positive NSCLC, with ECOG score of 0–2, with no prior systemic treatment	Crizotinib 250 mg BID (151) Alectinib 600 mg BID (152)	17.6 (0.3 to 27.0) 18.6 (0.5 to 29.0)	No	TR death; OS; PFS (investigator assessed)*	Phama
Hida 2017, p. 29[21] (J-ALEX; JAPICcti-132316)	≥ 20 yr, ALK-positive NSCLC, with ECOG score of 0–2, ALK-inhibitor naive, chemotherapy-naïve or had received 1 regimen of chemotherapy	Crizotinib 250 mg BID (104) Alectinib 300 mg BID (103)	NR	Not during study period; Treatment crossover after study withdrawal was allowed in both groups	TR death; PFS (independent review)*	Pharma
Hida 2016, p. 1642 (JP28927; JapicCTI-132186)[19] (Nishio 2018[42])	≥ 20 yr, ALK-positive NSCLC, with ECOG score of 0–1; prior treatment, including other ALK inhibitors, was allowed	Cross-over (300 mg BID total for all groups; 35 participants): Alectinib 20/40 mg capsules Alectinib 150 mg capsules Extension: Alectanib 300 mg BID (150 mg capsules)	13.1 (range 11.1 to 15.0)	Yes by design during cross-over phase	TR death	Pharma
Kim 2017 (ALTA, NCT02094573)[22, 43] (Kawata 2019[44])	≥ 18 yr, ALK-positive NSCLC, with ECOG performance status of 0–2, disease progression while receiving crizotinib	Brigatinib 90 mg QD (109) Brigatinib 180 mg QD (110)	NR	Yes, participants in the 90 mg/d group could cross to the 180 mg/d group after disease progression	PFS (independent review), SAEs	Pharma

BID = twice daily, ECOG = Eastern Cooperative Oncology Group, NSCLC = non-small cell lung cancer, OS = overall survival, PFS = progression-free survival, QD = once daily, RCT = randomized controlled trial, SAE = serious adverse event, TR = treatment-related, WHO = World Health Organization.

*Primary outcome.

The median age of participants across RCTs ranged from 45 to 61 years (Table 2). Studies were relatively balanced in terms of sex (37% to 64% male) and ECOG status, and most studies predominantly enrolled participants with no history of smoking (46%–75%) and who had adenocarcinoma (90%–100%), although one small RCT[18] enrolled a higher proportion of participants with squamous NSCLC (64%).

**Table 2:** Participants characteristics of included randomized controlled trials.

Author, yr, page (study name; NCT no.)	Group	Age, yr, median (range)*	Male, %	Current smoking,%	Never smoked,%	Brain or CNS metastases, %	ECOG0, %	ECOG 1, %	ECOG2, %	Adenocarcinoma, %
**Treatment naive**										
Zhou 2019[29] (ALESIA; NCT02838420)	Crizotinib	49 (IQR 41–59)	55	5	73	37	98**		2	97
	Alectinib	51 (IQR 43–59)	51	3	67	35	97**		3	94
Wu 2018[28] (PROFILE 1029; NCT01639001)	Chemotherapy	50 (23–69)	42	9	70	31	96**		4	98
	Crizotinib	48 (24–67)	48	7	75	20	96**		4	96
Camidge 2018[25] (ALTA-1L; NCT02737501)	Crizotinib	60 (29–89)	41	5	54	30	96**		4	99
	Brigatinib	58 (27–86)	50	3	61	29	96**		4	92
Soria 2017, p. 917 (ASCEND-4; NCT01828099)	Chemotherapy	54.0 (22–80)	39	8	65	33	37†	56†	6†	98
	Ceritinib	55.0 (22–81)	46	8	57	31	37	57	7	95
Peters 2017[5] (ALEX; NCT02075840)	Crizotinib	54.0 (18–91)	42	3	65	38	93**		7	94
	Alectinib	58.0 (25–88)	45	8	61	42	93**		7	90
Solomon 2014[17] (PROFILE 1014; NCT01154140)	Chemotherapy	54 (19–78)	37	3	65	27	95**		5	94
	Crizotinib	52 (22–76)	40	6	62	26	94**		6	94
**Treatment experienced**										
Novello 2018[26] (ALUR; NCT02604342)	Chemotherapy	59 (37–80)	49	6	46	74	31	54	14	100
	Alectinib	55.5 (21, 82)	57	3	49	65	40	51	8	100
Hida 2017[21] (J-ALEX; JAPICcti-132316)	Crizotinib	59.5 (25–84)	39	3	59	28	46	52	2	99
	Alectinib	61.0 (27–85)	40	2	54	14	52	46	2	97
Kim 2017[22] (ALTA; NCT02094573)	BRI 90 QD	50.5 (18–82)	45	NR	63	71	30	63	6	96
	BRI 180 QD	56.5 (20–81)	42	NR	57	67	41	51	8	98
Shaw 2017[23] (ASCEND-5; NCT01828112)	Chemotherapy	54.0 (47.0–64.0)¶	47	1	53	59	44†	52†	4†	97
	Ceritinib	54.0 (44.0–63.0)¶	41	3	62	57	49	43	8	97
Hida 2016[19] (JP28927; JapicCTI-132186)	Alectinib (cross-over)	45.0 (21–78)	46	3	60	NR	43	57	NR	100
Zhao 2015[18]	Chemotherapy	58.1 (13.2)‡	64	NR	NR	NR	NR	NR	NR	29
	Crizotinib	55.3 (12.7)‡	57	NR	NR	NR	NR	NR	NR	43
Shaw 2013[16] (PROFILE 1007; NCT00932893)	Chemotherapy	49 (24–85)	45	5	64	34	37	55	8	94
	Crizotinib	51 (22–81)	43	3	62	35	42	49	9	95

BRI = brigatinib, CNS = central nervous system, ECOG = Eastern Cooperative Oncology Group, IQR = interquartile range, NR = not reported, QD = once daily, SD = standard deviation.

*Unless otherwise stated.

^†^WHO performance score.

^‡^Mean (SD).

^¶^Median (IQR).

**ECOG0 or ECOG1.

### Risk of bias

Most RCTs were at low ROB for randomization (62%) and allocation concealment (54%), although 38% and 46% of studies did not report details of randomization and allocation concealment, respectively (S1 File Appendix 4). Performance and detection bias were of concern for all RCTs because of the open-label design. All RCTs that reported progression-free survival employed an independent review committee to ascertain disease progression; however, the primary outcome in three RCTs[5, 26, 29] was based on unblinded assessment of progression-free survival by trial investigators. The ROB owing to selective reporting was unclear for 23% of RCTs, primarily owing to a lack of available protocol or registration record; two RCTs[25, 29] (15%) were at high ROB owing to differences between the protocol and published manuscript. Other concerns included the potential for participant cross-over between study groups with unclear reporting of outcome data by group allocation.

### Synthesis of results

The evidence base for this review was formed by 13 RCTs[5, 16–19, 21–26, 28, 29] that reported at least one outcome of interest. Network meta-analyses were performed for progression-free survival, overall survival, and SAEs; treatment-related deaths were infrequently reported, and data were insufficient for NMA. Based on clinical similarity of populations across RCTs, and supported by the diagnostic considerations (S1 File Appendix 5), the fixed-effects model was deemed an appropriate fit for all outcomes. Additionally, the consistency model was a better fit for the data than the inconsistency model. We were unable to assess publication bias owing to the low number of studies included for each outcome. A summary of all analyses is available in Table 3.

**Table 3:** Summary of analyses.

Outcome	Meta-analysis (class effect v. chemotherapy)				Network-meta-analysis (effect of individual ALK inhibitors)			
	No. of RCTs*	No. of participants	Effect estimate (95%CI); *I*^2^	Finding	No. of RCTs	No. of participants	No. of comparisons	Finding
**Treatment-related death**	6†	1508	OR 2.58 (0.76 to 11.37), RD 0.49 (–0.16 to 1.46); 0%	• No difference in risk between crizotinib and chemotherapy; no treatment-related deaths reported for other ALK inhibitors	—	—	—	—
**Overall survival**	6	1611	HR 0.84 (0.72 to 0.97); 0%	• ALK inhibitors improved OS relative to chemotherapy	9	2376	9	• Alectinib improved overall survival relative to chemotherapy and crizotinib; no statistically significant difference between chemotherapy and crizotinib, ceritinib, or brigatinib
**Progression-free survival**	6	1611	HR 0.47 (0.41 to 0.53); 0%	• ALK inhibitors improved PFS relative to chemotherapy	10	2583	10	Crizotinib, ceritinib, alectinib, and brigatinib were significantly better than chemotherapy Alectinib and brigatinib were significantly better than crizotinib and ceritinib
**Serious adverse events**	6	1584	OR 1.67 (1.34 to 2.08); 62%	• ALK inhibitors increased the risk of SAE relative to chemotherapy	8	2074	8	Risk of SAEs was significantly higher with crizotinib and alectinib compared with chemotherapy Risk of SAEs was lower with ceritinib than with crizotinib

HR = hazard ratio, OR = odds ratio, OS = overall survival, PFS = progression-free survival, RCT = randomized controlled trial, RD = risk difference, SAE = serious adverse event

*RCTs that involved an ALK inhibitor compared to chemotherapy.

^†^Six chemotherapy-controlled RCTs reported 6 treatment-related deaths among patients who received crizotinib. An additional 4 treatment-related deaths were reported among those exposed to crizotinib in head-to-head RCTs of different ALK inhibitors. See S1 File for full details.

#### Treatment-related deaths

Eleven treatment-related deaths were reported in 5 RCTs[5, 16, 17, 28, 29]; an additional 6 RCTs[18, 19, 21, 23–25] reported that no treatment-related deaths had occurred (S1 File Appendix 6). Of the 11 reported deaths, 10 occurred among participants who had received crizotinib (n = 917); one death was deemed related to chemotherapy (n = 765). No deaths were related to ceritinib (n = 304), alectinib (n = 415), or brigatinib (n = 137); however, the duration of treatment and follow-up was not consistent across ALK inhibitors (S1 File Appendix 6). Of the 10 crizotinib-related deaths, the causes of 4 deaths were not reported; 5 deaths were attributed to pneumonitis or interstitial lung disease and 1 to arrhythmia. Compared with chemotherapy, there was no statistically significant difference in the risk of death between crizotinib and chemotherapy (OR 2.59 [95%CrI 0.76 to 11.37]; RD 0.49 [95%CrI –0.16 to 1.46]; *I*^2^ = 0%) when assessed via pairwise meta-analysis. Similarly, there was no statistically significant difference between treatment naive (OR 2.59 [95%CrI 0.76 to 11.37]; *I*^2^ = 0%) or experienced participants (OR 2.23 [95% CrI 0.40 to 19.66]; *I*^2^ = 0) (p for subgroups = 0.87). Relative risks are reported in S1 File Appendix 6.

#### Overall survival

Nine RCTs[5, 16, 17, 23–26, 28, 29] assessed overall survival (S1 File Appendix 7). Significant improvement in overall survival was not confirmed in any of these trials, with the exception of a statistically significant improvement in overall survival with alectinib over crizotinib in the ALESIA trial[29]; however, the findings of other studies may have been affected by confounding by participant crossover between study arms. When we assessed overall survival with any ALK inhibitor relative to chemotherapy via pairwise meta-analysis, treatment with any ALK inhibitor improved overall survival relative to chemotherapy (HR 0.84, 95%CrI 0.72–0.97; n = 1611; *I*^2^ = 0%; 6 RCTs[16, 17, 23, 24, 26, 28]); this difference was conserved among treatment-naive (HR 0.78, 95%CrI 0.62–0.97; *I*^2^ = 0%;) but not experienced (HR 0.90, 95%CrI 0.73–1.11; *I*^2^ = 0%) participants (p for subgroup differences p = 0.36). However, rate of crossover from chemotherapy to ALK inhibitor was high in some studies or was unreported but potentially high in others. Of note, the published overall survival data remain immature for several RCTs (S1 File Appendix 7). The 12-month survival rate ranged from 70% to 86% for ALK inhibitors and 67% to 79% for chemotherapy.

**Fig 2:**
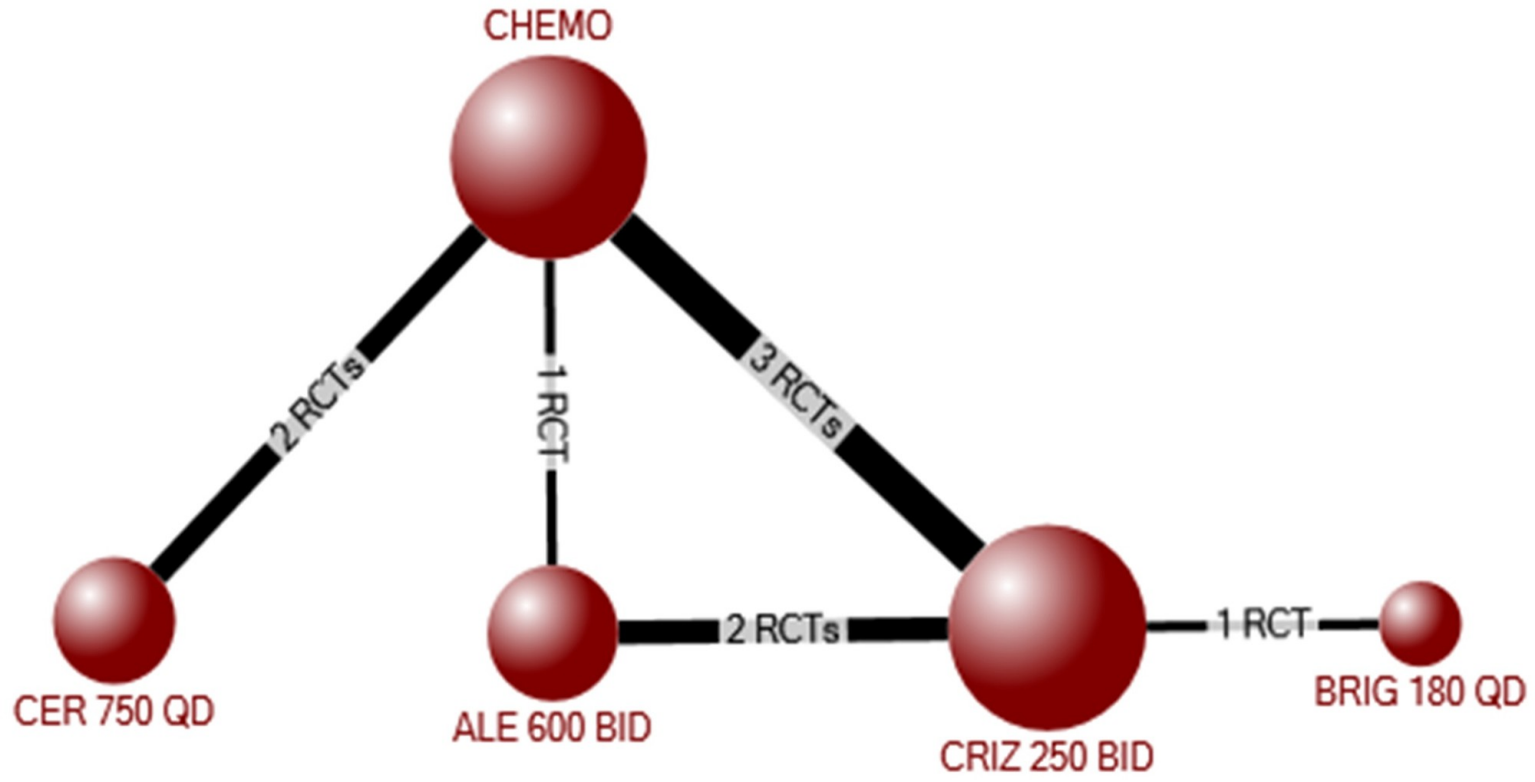
Evidence network for the network meta-analysis of overall survival among all participants (treatment experienced and naïve).

Next, we considered whether there was a differential effect of individual ALK inhibitors on overall survival via network meta-analysis. The evidence network for overall survival (based on HRs) included 2376 participants randomized to crizotinib, ceritinib, alectinib, brigatinib, or chemotherapy in 9 RCTs[5, 16, 17, 23–26, 28, 29] (Fig 2). In the NMA, alectinib was significantly better than both chemotherapy (HR 0.57, 95%CrI 0.39–0.83) and crizotinib (HR 0.68, 95%CrI 0.48–0.96), with no other statistically significant differences between the other ALK inhibitors (Table 4). The results of pair-wise meta-analyses for each combination of treatments included in the network is shown in S1 File Appendix 7B. The findings were similar among treatment naive, but not experienced, participants (S1 File Appendix 7C), although the impact of crossover would again be expected to be high.

**Table 4:** Network meta-analysis of hazard ratios for overall survival for individual ALK inhibitors among all patients (experienced and naïve) with ALK-positive non-small cell lung cancer.

	Hazard ratio (95% credible interval)*			
	CHEMO	CRIZ 250 BID	CER 750 QD	ALE 600 BID
**CRIZ 250 BID**	0.84 (0.70, 1.01)	—		
**CER 750 QD**	0.85 (0.64, 1.13)	1.01 (0.73, 1.41)	—	
**ALE 600 BID**	**0.57 (0.39, 0.83)**	**0.68 (0.48, 0.96)**	0.67 (0.42, 1.07)	—
**BRIG 180 QD**	0.82 (0.41, 1.65)	0.98 (0.50, 1.91)	0.97 (0.46, 2.02)	1.44 (0.68, 3.08)

ALE = alectinib, BID = twice daily, BRIG = brigatinib, CER = ceritinib, CHEMO = chemotherapy, CRIZ = crizotinib, QD = once daily.

*Fixed-effects model. Significant changes are indicated by use of bold and colour (green indicates that the row treatment is significantly better than the column treatment). White indicates no significant difference between treatments.

#### Progression-free survival

In total, 12 RCTs[5, 16–18, 21–26, 28, 29] assessed progression-free survival, as time to progression (10 RCTS[5, 16, 17, 21, 23–26, 28, 29]) and/or the percentage progression-free survival at 12 months (6 RCTs[5, 16, 22–25]). Among chemotherapy-controlled trials, six RCTs[5, 17, 24, 25, 28, 29] involved treatment-naive participants (n = 926) and three RCTs[16, 23, 26] involved treatment-experienced participants (n = 685) (S1 File Appendix 8). Compared with chemotherapy via meta-analysis, treatment with any ALK inhibitor significantly improved progression-free survival among all participants (HR 0.47, 95%CrI 0.41–0.53, *I*^2^ = 0%), with similar results among both treatment-experienced (HR 0.47, 95%CrI 0.39–0.57; *I*^2^ = 0%) and naive participants (HR 0.47, 95%CrI 0.40–0.56; *I*^2^ = 0%) (p for subgroups 0.99). The percentage of participants who were progression free at 12 months ranged from 18% to 68% among participants who received an ALK inhibitor, and between 6% and 39% among those who received chemotherapy.

**Fig 3:**
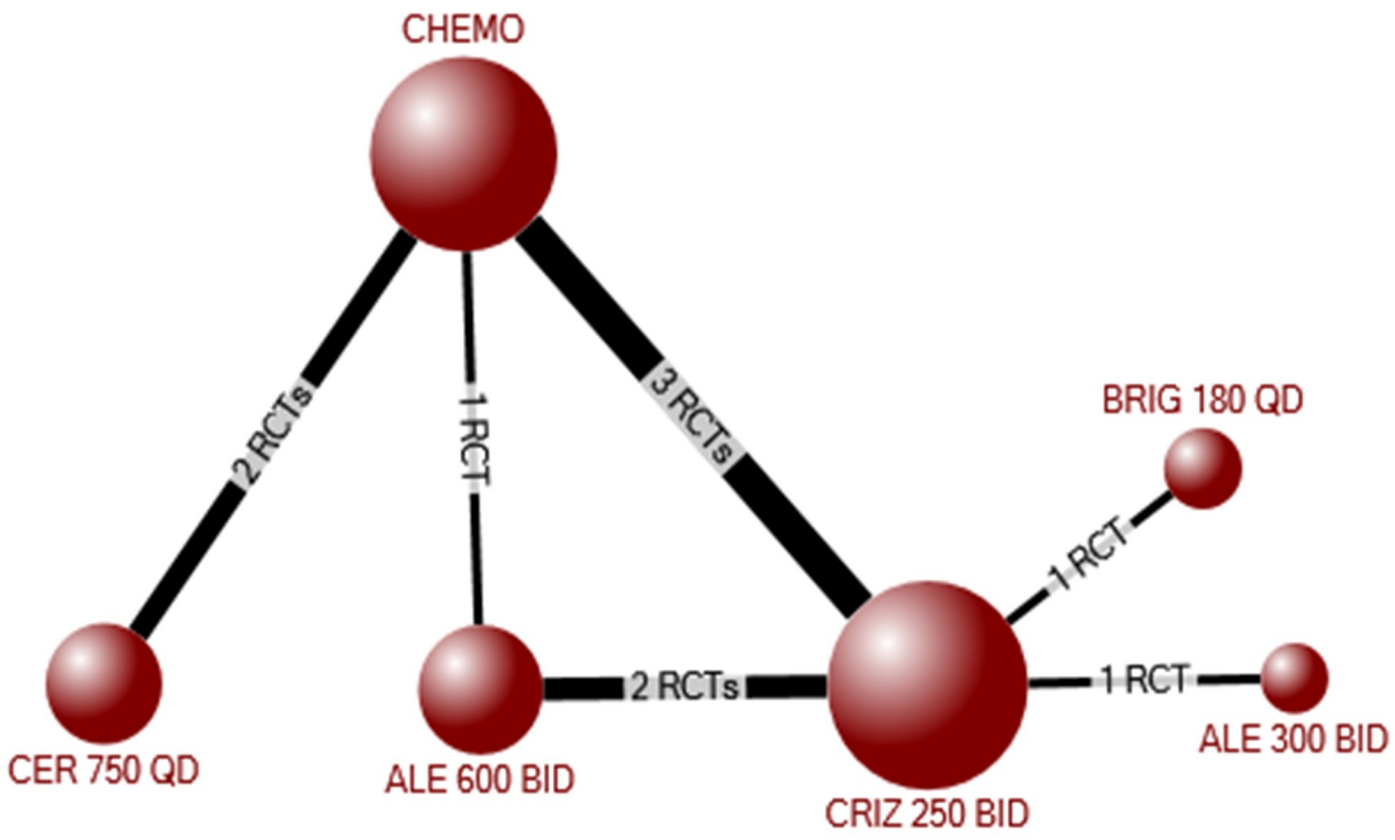
Evidence network for the network meta-analysis of progression-free survival among all participants (treatment experienced and naïve).

The network meta-analysis for progression-free survival included 2583 participants randomized to crizotinib, ceritinib, alectinib, brigatinib, or chemotherapy in 10 RCTs[5, 16, 17, 21, 23–26, 28, 29] (Fig 3). Compared with placebo, each individual ALK inhibitor improved progression-free survival (crizotinib: HR 0.46, 95%CrI 0.39–0.54; ceritinib: HR 0.52, 95%CrI 0.42–0.64; alectinib 300 BID: 0.16, 95%CrI 0.08–0.33; alectinib 600 mg BID: 0.23, 95%CrI 0.17–0.30; brigatinib: HR 0.23, 95%CrI 0.15–0.35) (Table 5). Among the ALK inhibitors, there was no difference in progression-free survival between ceritinib and crizotinib, between alectinib and brigatinib, or between doses of alectinib (300 v. 600 mg BID). However, alectinib and brigatinib were both significantly better than crizotinib (alectinib 300 BID: HR 0.34, 95%CrI 0.17–0.70; alectinib 600 BID: HR 0.49, 95%CrI 0.38–0.63; brigatinib: HR 0.49, 95%CrI 0.33–0.73) and ceritinib (alectinib 300 BID: HR 0.30, 95%CrI 0.14–0.64; alectinib 600 BID: HR 0.43, 95%CrI 0.31–0.62; brigatinib: HR 0.43, 95%CrI 0.27–0.70) (Table 5). The results of pair-wise meta-analyses for each combination of treatments included in the network is shown in S1 File Appendix 8B. The results were similar among participants with previous treatment experience or no previous experience, although there was no statistically significant difference between alectinib 300 BID and crizotinib or ceritinib among treatment-experienced participants (S1 File Appendix 8).

**Table 5:** Network meta-analysis of hazard ratios for progression-free survival for individual ALK inhibitors among all patients (experienced and naïve) with ALK-positive non-small cell lung cancer.

	Hazard ratio (95% credible interval)*					
	CHEMO	CRIZ 250 BID	CER 750 QD	ALE 300 BID	ALE 600 BID	BRIG 180 QD
**CRIZ 250 BID**	**0.46 (0.39, 0.54)**	—				
**CER 750 QD**	**0.52 (0.42, 0.64)**	1.13 (0.87, 1.47)	—			
**ALE 300 BID**	**0.16 (0.08, 0.33)**	**0.34 (0.17, 0.70)**	**0.30 (0.14, 0.64)**	—		
**ALE 600 BID**	**0.23 (0.17, 0.30)**	**0.49 (0.38, 0.63)**	**0.43 (0.31, 0.62)**	1.44 (0.67, 3.05)	—	
**BRIG 180 QD**	**0.23 (0.15, 0.35)**	**0.49 (0.33, 0.73)**	**0.43 (0.27, 0.70)**	1.44 (0.63, 3.25)	1.00 (0.62, 1.61)	—

ALE = alectinib, BID = twice daily, BRIG = brigatinib, CER = ceritinib, CHEMO = chemotherapy, CRIZ = crizotinib, QD = once daily.

*Fixed-effects model. Significant changes are indicated by use of bold and colour (green indicates that the row treatment is significantly better than the column treatment). White indicates no significant difference between treatments.

#### Serious adverse events

Nine RCTs[5, 16, 17, 22–24, 26, 28, 29] reported SAEs occurring with ALK treatment or chemotherapy (S1 File Appendix 9). Compared with chemotherapy via meta-analysis, the use of any ALK inhibitor was associated with an increased risk of SAEs (OR 1.67 [95%CrI 1.34–2.08]; *I*^2^ = 62%) among all patients. The results were consistent among both treatment experienced (OR 1.75 [95%CrI 1.23–2.46]; *I*^2^ = 73%) and naive participants (OR 1.42 [95%CrI 1.10–1.89]; *I*^2^ = 18%) (p value for subgroup effect = 0.12). Relative risks are reported in S1 File Appendix 9.

The network meta-analysis for SAEs included 2074 participants randomized to crizotinib, ceritinib, alectinib, or chemotherapy in 8 RCTs.[5, 16, 17, 23, 24, 26, 28, 29] One RCT[22] involving brigatinib could not be included in the network because of a lack of common comparators. The network diagram is shown in S1 File Appendix 9. Compared with chemotherapy, crizotinib and alectinib, but not ceritinib, were associated with an increased risk of an SAE (crizotinib: OR 2.08, 95%CrI 1.56–2.79; alectinib: OR 1.60, 95%CI 1.00–2.58) (RRs are available in S1 File Appendix 9). Among the ALK inhibitors, ceritinib was associated with fewer SAEs compared with crizotinib (OR 0.60, 95%CrI 0.39–0.93); there were no other statistically significant differences between crizotinib and alectinib or between ceritinib and alectinib. The results of pair-wise meta-analyses for each combination of treatments included in the network is shown in S1 File Appendix 9C. When examined by treatment-history, crizotinib was associated with more SAEs compared with chemotherapy among both treatment-naive and -experienced participants (S1 File Appendix 9).

## Discussion

Treatment-related deaths were infrequent among participants who received an ALK inhibitor, with no statistically significant difference in risk between crizotinib and chemotherapy; of note, no treatment-related deaths were reported for any other ALK inhibitor. In the NMA, we observed that all ALK inhibitors improved progression-free survival relative to chemotherapy, and that alectinib and brigatinib were associated with improved progression-free survival compared to crizotinib and ceritinib.

In terms of overall survival, alectinib improved overall survival relative to chemotherapy and crizotinib, although the findings may have been influenced by crossover between treatment groups following disease progression. The net effect of such crossovers may be that the overall survival of patients receiving an ALK inhibitor is substantially longer than that of patients who received chemotherapy alone. This discrepancy is emphasized by differences in the duration of treatment exposure between patients randomized to chemotherapy and ALK inhibitors among RCTs that allowed cross-over. For example, in the ASCEND-5 trial,[23] which permitted crossover of patients from the chemotherapy arm to the ceritinib arm after progression, the median treatment exposure was about 30 weeks for ceritinib compared with 6 weeks for chemotherapy. This is consistent with clinical experience which suggests that ALK inhibitors are effective in patients with previous treatment experience. Indeed, longer progression-free survival, as well as improved quality of life, has been reported with crizotinib after disease progression on chemotherapy (PROFILE 1007[16]), as well as with ceritinib after progression on chemotherapy or crizotinib (ASCEND-5[23]), despite a lack of statistically significant differences in overall survival.

There exists limited clinical trial data directly comparing the efficacy of individual ALK inhibitors. Three RCTs have directly compared crizotinib and alectinib in either treatment-naïve (ALEX[5], ALESIA[29]) or -experienced (J-ALEX[21]) participants; in each, alectinib significantly improved progression-free survival compared with crizotinib. Data for overall survival remain immature, but may be improved with alectinib.[29] Similarly, in a recent head-to-head trial (ALTA-1L)[25] brigatinib improved progression-free survival compared with crizotinib, although there was no statistically significant difference in overall survival. The findings from our NMA comparing the effects of individual ALK inhibitors are consistent with these RCTs: we found that alectinib and brigatinib significantly improved progression-free survival relative to crizotinib. Additionally, we found that alectinib and brigatinib were both more effective than ceritinib at improving progression-free survival; these two ALK inhibitors have yet to be directly compared in a head-to-head RCT. Consistent with this, guidelines by the European Society of Medical Oncology[45] have been recently revised to include alectinib and brigatinib as a potential treatments for *ALK*-positive NSCLC.

In this review, use of crizotinib (but not ceritinib or alectinib) was associated with an increased risk of SAEs. In the two head-to-head trials of crizotinib and chemotherapy (PROFILE 1007,[16] PROFILE 1014[17]), these were driven largely by elevated levels of aminotransferase, which may be managed by a break from treatment and a reduction in dose. Results from PROFILE1007 suggest that there may be no important difference in the incidence of treatment-related SAEs (crizotinib:12%; chemotherapy:14%) and no difference in treatment-related adverse events leading to permanent discontinuation of the study drug (crizotinib:6%; chemotherapy:10%).[16] This finding was echoed in PROFILE 1014, with 5% and 8% of participants permanently discontinuing treatment because of treatment-related adverse events.[17]

Clinical evidence for the treatment of NSCLC with *ROS1* rearrangements lags behind that for *ALK*-positive NSCLC. In this review, we found no RCTs involving patients with *ROS1* rearrangements. Few prospective non-randomized studies have assessed outcomes with the use of ALK inhibitors in this population, although several small single-arm phase II studies have reported 12 month survival of 83%–85% (median progression-free survival of at least 15 months) with crizotinib.[46–48] Current recommendations from the American Society of Clinical Oncology[49] and the European Society of Medical Oncology[45] support the use of crizotinib as first-line treatment for patients with *ROS1* rearrangements; additional research is needed to assess the benefits of other ALK inhibitors in this population.

### Strengths and limitations

We performed a comprehensive search of the published and grey literature for randomized and non-randomized studies, without language or date restrictions, and the protocol was registered a priori. We used NMA methodology to assess the relative efficacy and safety of individual ALK inhibitors. Previous reviews have used pair-wise meta-analysis to compare two treatments at a time or to pool the percentage of patients with an outcome from single-arm non-randomized studies.[6, 50–52] While such analyses are important initial steps, they provide limited evidence to inform clinical practice, because clinicians must select between all available treatments, not between pairs of isolated treatments.

Our study has several limitations that merit consideration. First, we did not have access to individual participant data, and we are limited to describing the number and causes of deaths as reported in the published studies. Treatment-related deaths are uncommon with these agents, and the included RCTs may have been underpowered to detect differences in treatment-related deaths and other safety outcomes.

Second, cause of death was not well reported, and the classification of a death as “treatment-related” was at the discretion of the investigators. As well, the duration of treatment varied between the RCTs, with participants exposed to ALK inhibitors for considerably longer than chemotherapy, likely owing to crossover to an ALK inhibitor after disease progression with chemotherapy. This has important implications for the assessment of several outcomes, including treatment-related deaths, overall survival, and SAEs, and the results should be interpreted with caution.

Third, there are limitations associated with the outcomes progression-free and overall survival. Progression-free survival may be considered as a surrogate marker for overall survival, but it has not been validated as such.[53] Overall survival is the most objective outcome with which to assess efficacy of cancer treatments; however, its measurement is confounded by the changing of treatments after disease progression.[53] Most RCTs allowed participants receiving chemotherapy to switch to an ALK inhibitor after disease progression, and we were unable to analyze the data separately for those who did or did not switch treatments. Progression-free survival is not affected by such treatment switches; however, it may be prone to measurement error and bias, and it does not capture the entire treatment effect on outcomes that are important to participants (e.g., prolonged survival, quality of life).[53] Along with a longer duration of progression-free survival, improved quality of life was noted in several RCTs with the use of ALK inhibitors, suggesting that extending the period before tumour progression will be of value to patients.

Fourth, relatively few RCTs have assessed the efficacy of ALK inhibitors. As such, our analyses comparing the effects of individual ALK inhibitors involved few trials, and the findings may change with the publication of future trials. We were unable to assess publication bias because few studies contributed data to each outcome. Similarly, we were unable to fully assess the impact of patient characteristics on the outcomes, and future studies should consider other important variables such as age and sex, as well as the influence of cross-over between treatment groups.

## Conclusion

Treatment-related deaths were infrequent among *ALK*-positive NSCLC. Among patients with *ALK*-positive NSCLC, progression-free survival was improved by crizotinib, ceritinib, alectinib, and brigatinib compared with chemotherapy, while alectinib and brigatinib were significantly better than crizotinib and ceritinib. Overall survival was improved only by alectinib; however, the findings are likely confounded by crossover between treatment groups and should be interpreted with caution. Few studies have enrolled participants with *ROS1* mutations, and additional research is need in this area.

## Supporting information

S1 File Supplementary online content (Appendices 1–9). Appendix 1: PRISMA NMA Checklist. Appendix 2: Search strategy. Appendix 3: Included studies. Appendix 4: Risk of bias. Appendix 5: Model diagnostics. Appendix 6: Treatment-related death. Appendix 7: Overall survival. Appendix 8: Progression-free survival. Appendix 9: Serious adverse events.
(DOCX)
