# Supplementary material for: ALK inhibitors for non-small cell lung cancer: A systematic review and network meta-analysis
Source: PLoS One. 2020 Feb 19;15(2):e0229179. doi: 10.1371/journal.pone.0229179 (PMC7029857; doi:10.1371/journal.pone.0229179)
Supplement: S1 File — Appendix 1: PRISMA NMA Checklist. Appendix 2: Search strategy. Appendix 3: Included studies. Appendix 4: Risk of bias. Appendix 5: Model diagnostics. Appendix 6: Treatment-related death. Appendix 7: Overall survival. Appendix 8: Progression-free survival. Appendix 9: Serious adverse events. (DOCX) [file pone.0229179.s001.docx]

**Supplemental File**

Contents

[Appendix 1. PRISMA NMA Checklist 2](#_Toc28865297)

[Appendix 2. Search strategy 5](#_Toc28865298)

[Appendix 3. Included studies 10](#_Toc28865299)

[Appendix 4. Risk of bias assessment 12](#_Toc28865300)

[Appendix 5: Model diagnostics 23](#_Toc28865301)

[Appendix 6. Treatment-related death 25](#_Toc28865302)

[Appendix 7. Overall survival 26](#_Toc28865303)

[Appendix 8. Progression-free survival 29](#_Toc28865304)

[Appendix 9. Serious adverse events 32](#_Toc28865305)

# Appendix 1. PRISMA NMA checklist

| **Section/Topic** | **Item #** | **Checklist Item** | **Reported on Page #** |
| --- | --- | --- | --- |
| **TITLE** |  |  |  |
| Title | 1 | Identify the report as a systematic review *incorporating a network meta-analysis (or related form of meta-analysis).* | 1 |
| **ABSTRACT** |  |  |  |
| Structured summary | 2 | Provide a structured summary including, as applicable:  **Background:** main objectives  **Methods:** data sources; study eligibility criteria, participants, and interventions; study appraisal; and *synthesis methods, such as network meta-analysis.*  **Results:** number of studies and participants identified; summary estimates with corresponding confidence/credible intervals; *treatment rankings may also be discussed. Authors may choose to summarize pairwise comparisons against a chosen treatment included in their analyses for brevity.*  **Discussion/Conclusions:** limitations; conclusions and implications of findings.  **Other:** primary source of funding; systematic review registration number with registry name. | 2 |
| **INTRODUCTION** |  |  |  |
| Rationale | 3 | Describe the rationale for the review in the context of what is already known*, including mention of why a network meta-analysis has been conducted.* | 7 |
| Objectives | 4 | Provide an explicit statement of questions being addressed, with reference to participants, interventions, comparisons, outcomes, and study design (PICOS). | 7 |
| **METHODS** |  |  |  |
| Protocol and registration | 5 | Indicate whether a review protocol exists and if and where it can be accessed (e.g., Web address); and, if available, provide registration information, including registration number. | 8 |
| Eligibility criteria | 6 | Specify study characteristics (e.g., PICOS, length of follow-up) and report characteristics (e.g., years considered, language, publication status) used as criteria for eligibility, giving rationale. *Clearly describe eligible treatments included in the treatment network, and note whether any have been clustered or merged into the same node (with justification).* | 8 |
| Information sources | 7 | Describe all information sources (e.g., databases with dates of coverage, contact with study authors to identify additional studies) in the search and date last searched. | 8 |
| Search | 8 | Present full electronic search strategy for at least one database, including any limits used, such that it could be repeated. | Appendix |
| Study selection | 9 | State the process for selecting studies (i.e., screening, eligibility, included in systematic review, and, if applicable, included in the meta-analysis). | 8 |
| Data collection process | 10 | Describe method of data extraction from reports (e.g., piloted forms, independently, in duplicate) and any processes for obtaining and confirming data from investigators. | 9 |
| Data items | 11 | List and define all variables for which data were sought (e.g., PICOS, funding sources) and any assumptions and simplifications made. | 9 |
| **Geometry of the network** | **S1** | Describe methods used to explore the geometry of the treatment network under study and potential biases related to it. This should include how the evidence base has been graphically summarized for presentation, and what characteristics were compiled and used to describe the evidence base to readers. | 9-10 |
| Risk of bias within individual studies | 12 | Describe methods used for assessing risk of bias of individual studies (including specification of whether this was done at the study or outcome level), and how this information is to be used in any data synthesis. | 9 |
| Summary measures | 13 | State the principal summary measures (e.g., risk ratio, difference in means). *Also describe the use of additional summary measures assessed, such as treatment rankings and surface under the cumulative ranking curve (SUCRA) values, as well as modified approaches used to present summary findings from meta-analyses.* | 10 |
| Planned methods of analysis | 14 | Describe the methods of handling data and combining results of studies for each network meta-analysis. This should include, but not be limited to:   - *Handling of multi-arm trials;* - *Selection of variance structure;* - *Selection of prior distributions in Bayesian analyses; and* - *Assessment of model fit.* | 10 |
| **Assessment of Inconsistency** | **S2** | Describe the statistical methods used to evaluate the agreement of direct and indirect evidence in the treatment network(s) studied. Describe efforts taken to address its presence when found. | 10 |
| Risk of bias across studies | 15 | Specify any assessment of risk of bias that may affect the cumulative evidence (e.g., publication bias, selective reporting within studies). | 10 |
| Additional analyses | 16 | Describe methods of additional analyses if done, indicating which were pre-specified. This may include, but not be limited to, the following:   - Sensitivity or subgroup analyses; - Meta-regression analyses; - *Alternative formulations of the treatment network; and* - *Use of alternative prior distributions for Bayesian analyses (if applicable).* | 10 |
| **RESULTS†** |  |  |  |
| Study selection | 17 | Give numbers of studies screened, assessed for eligibility, and included in the review, with reasons for exclusions at each stage, ideally with a flow diagram. | 11, Figure 1 |
| **Presentation of network structure** | **S3** | Provide a network graph of the included studies to enable visualization of the geometry of the treatment network. | Figure 4 |
| **Summary of network geometry** | **S4** | Provide a brief overview of characteristics of the treatment network. This may include commentary on the abundance of trials and randomized patients for the different interventions and pairwise comparisons in the network, gaps of evidence in the treatment network, and potential biases reflected by the network structure. | 12-15, by outcome |
| Study characteristics | 18 | For each study, present characteristics for which data were extracted (e.g., study size, PICOS, follow-up period) and provide the citations. | Table 1 |
| Risk of bias within studies | 19 | Present data on risk of bias of each study and, if available, any outcome level assessment. | Appendix 4 |
| Results of individual studies | 20 | For all outcomes considered (benefits or harms), present, for each study: 1) simple summary data for each intervention group, and 2) effect estimates and confidence intervals. *Modified approaches may be needed to deal with information from larger networks.* | 12-15 |
| Synthesis of results | 21 | Present results of each meta-analysis done, including confidence/credible intervals. *In larger networks, authors may focus on comparisons versus a particular comparator (e.g. placebo or standard care), with full findings presented in an appendix. League tables and forest plots may be considered to summarize pairwise comparisons.* If additional summary measures were explored (such as treatment rankings), these should also be presented. | 12-15 |
| **Exploration for inconsistency** | **S5** | Describe results from investigations of inconsistency. This may include such information as measures of model fit to compare consistency and inconsistency models, *P* values from statistical tests, or summary of inconsistency estimates from different parts of the treatment network. | NA, p. 11 |
| Risk of bias across studies | 22 | Present results of any assessment of risk of bias across studies for the evidence base being studied. | 12 |
| Results of additional analyses | 23 | Give results of additional analyses, if done (e.g., sensitivity or subgroup analyses, meta-regression analyses*, alternative network geometries studied, alternative choice of prior distributions for Bayesian analyses,* and so forth). | 12-15, by outcome |
| **DISCUSSION** |  |  |  |
| Summary of evidence | 24 | Summarize the main findings, including the strength of evidence for each main outcome; consider their relevance to key groups (e.g., healthcare providers, users, and policy-makers). | 15-18 |
| Limitations | 25 | Discuss limitations at study and outcome level (e.g., risk of bias), and at review level (e.g., incomplete retrieval of identified research, reporting bias). *Comment on the validity of the assumptions, such as transitivity and consistency. Comment on any concerns regarding network geometry (e.g., avoidance of certain comparisons).* | 19 |
| Conclusions | 26 | Provide a general interpretation of the results in the context of other evidence, and implications for future research. | 20 |
| **FUNDING** |  |  |  |
| Funding | 27 | Describe sources of funding for the systematic review and other support (e.g., supply of data); role of funders for the systematic review. This should also include information regarding whether funding has been received from manufacturers of treatments in the network and/or whether some of the authors are content experts with professional conflicts of interest that could affect use of treatments in the network. | 3, 21 |

# Appendix 2. Search strategy

ALK Inhibitors – NSCLC

2017 June (Note: the search was subsequently updated July 23, 2019; number in parentheses represent numbers in the initial 2017 seach)

Note: The search strategy was designed identify randomized and non-randomized studies; non-randomized studies will be evaluated at a later date.

Ovid Multifile

Database: Embase <1974 to 2017 June 26>, Ovid MEDLINE(R) Epub Ahead of Print, In-Process & Other Non-Indexed Citations, Ovid MEDLINE(R) Daily and Ovid MEDLINE(R) <1946 to Present>

Search Strategy:

--------------------------------------------------------------------------------

1 Carcinoma, Non-Small-Cell Lung/ (44073)

2 non-small cell lung cancer*.tw,kw. (110361)

3 non-small cell lung carcinoma*.tw,kw. (10443)

4 nonsmall cell lung cancer*.tw,kw. (5615)

5 nonsmall cell lung carcinoma*.tw,kw. (997)

6 ((non-small cell cancer* or nonsmall cell cancer* or non-small cell carcinoma* or nonsmall cell carcinoma*) adj3 lung?).tw,kw. (1272)

7 NSCLC?.tw,kw. (88209)

8 non-small cell bronch* cancer*.tw,kw. (187)

9 non-small cell bronch* carcinoma*.tw,kw. (678)

10 nonsmall cell bronch* cancer*.tw,kw. (5)

11 nonsmall cell bronch* carcinoma*.tw,kw. (18)

12 ((non-small cell cancer* or nonsmall cell cancer* or non-small cell carcinoma* or nonsmall cell carcinoma*) adj3 bronch*).tw,kw. (63)

13 non-small cell pulmonary cancer*.tw,kw. (9)

14 non-small cell pulmonary carcinoma*.tw,kw. (34)

15 nonsmall cell pulmonary cancer*.tw,kw. (4)

16 nonsmall cell pulmonary carcinoma*.tw,kw. (0)

17 ((non-small cell cancer* or nonsmall cell cancer* or non-small cell carcinoma* or nonsmall cell carcinoma*) adj3 pulmonar*).tw,kw. (76)

18 ((lung? or bronch* or pulmonar*) adj1 (adenocarcinoma* or adeno-carcinoma* or adenocancer* or adeno-cancer*)).tw,kw. (31844)

19 large cell lung cancer*.tw,kw. (495)

20 large cell lung carcinoma*.tw,kw. (410)

21 ((large cell cancer* or large cell carcinoma*) adj3 (lung? or bronch* or pulmonar*)).tw,kw. (976)

22 (large cell neuroendocrine cancer* and (lung? or bronch* or pulmonar*)).tw,kw. (28)

23 (large cell neuroendocrine carcinoma* and (lung? or bronch* or pulmonar*)).tw,kw. (1566)

24 ((squamous cell cancer* or squamous cell carcinoma*) and (lung? or bronch* or pulmonar*)).tw,kw. (31839)

25 ((epidermoid cancer* or epidermoid carcinoma* or epidermoid cell cancer* or epidermoid cell carcinoma*) and (lung? or bronch* or pulmonar*)).tw,kw. (2001)

26 ((prickle cell adj (cancer* or carcinoma* or adenocarcinoma* or adeno-carcinoma* or adenocancer* or adeno-cancer*)) and (lung? or bronch* or pulmonar*)).tw,kw. (0)

27 (adenosquamous carcinoma* and (lung? or bronch* or pulmonar*)).tw,kw. (1284)

28 (sarcomatoid carcinoma* and (lung? or bronch* or pulmonar*)).tw,kw. (745)

29 (planocellular carcinoma* and (lung? or bronch* or pulmonar*)).tw,kw. (30)

30 or/1-29 [NSCLC] (191600)

31 (anaplastic lymphoma kinase adj3 inhibit*).tw,kw. (644)

32 (ALK adj3 inhibit*).tw,kw. (3399)

33 (ALKI or ALKIs).tw,kw. (52)

34 (crizotinib or "pf 02341066" or pf 1066 or pf 2341066 or pf02341066 or pf1066 or pf2341066 or xalkori).tw,kw,rn. (6919)

35 (ceritinib or ldk 378 or ldk378 or nvp ldk 378 or nvp ldk 378 nx or nvp ldk378 or nvp ldk378 nx or zykadia).tw,kw,rn. (1195)

36 (alectinib or af 802 or af802 or alecensa or alectinib hydrochloride or ch 5424802 or ch5424802 or rg 7853 or rg7853 or ro 5424802 or ro5424802).tw,kw,rn. (951)

37 (brigatinib or ap 26113 or ap26113).tw,kw,rn. (375)

38 (lorlaninib or "pf 06463922" or pf06463922).tw,kw,rn. (142)

39 (ASP3026 or ASP 3026 or UNII-HP4L6MXF10 or 1097917-15-1).tw,kw,rn. (125)

40 (X-396 or X396 or UNII-7DR7JMB8BH or 1365267-27-1).tw,kw,rn. (115)

41 (ensartinib or UNII-SMA5ZS5B22 or 370651-20-9).tw,kw,rn. (10)

42 (entrectinib or nms e 628 or nms e628 or rxdx 101 or rxdx101).tw,kw,rn. (170)

43 or/31-42 [ALK INHIBITORS] (8676)

44 30 and 43 [ALK INHIBITORS - NSCLC] (4678)

45 exp Animals/ not (exp Animals/ and Humans/) (14955509)

46 44 not 45 [ANIMAL-ONLY REMOVED] (2355)

47 limit 46 to systematic reviews [Limit not valid in Embase; records were retained] (957)

48 meta analysis.pt. (81934)

49 exp meta-analysis as topic/ (51504)

50 (meta-analy* or metanaly* or metaanaly* or met analy* or integrative research or integrative review* or integrative overview* or research integration or research overview* or collaborative review*).tw,kw. (275001)

51 (systematic review* or systematic overview* or evidence-based review* or evidence-based overview* or (evidence adj3 (review* or overview*)) or meta-review* or meta-overview* or meta-synthes* or rapid review* or "review of reviews" or technology assessment* or HTA or HTAs).tw,kw. (322301)

52 exp Technology assessment, biomedical/ (22294)

53 (cochrane or health technology assessment or evidence report).jw. (33279)

54 (network adj (MA or MAs)).tw,kw. (14)

55 (NMA or NMAs).tw,kw. (3561)

56 indirect comparison?.tw,kw. (3587)

57 (indirect treatment* adj1 comparison?).tw,kw. (463)

58 (mixed treatment* adj1 comparison?).tw,kw. (1140)

59 (multiple treatment* adj1 comparison?).tw,kw. (198)

60 (multi-treatment* adj1 comparison?).tw,kw. (3)

61 simultaneous comparison?.tw,kw. (863)

62 mixed comparison?.tw,kw. (34)

63 or/48-62 (581217)

64 46 and 63 (49)

65 47 or 64 [SYSTEMATIC REVIEWS] (967)

66 (controlled clinical trial or randomized controlled trial).pt. (556221)

67 clinical trials as topic.sh. (186967)

68 (randomi#ed or randomi#ation or randomly or RCT$1 or placebo*).tw. (1931897)

69 ((singl* or doubl* or trebl* or tripl*) adj (mask* or blind* or dumm*)).tw. (361131)

70 trial.ti. (412530)

71 or/66-70 (2391771)

72 46 and 71 [RCTS] (214)

73 controlled clinical trial.pt. (94240)

74 Controlled Clinical Trial/ or Controlled Clinical Trials as Topic/ (544185)

75 (control* adj2 trial*).tw. (481900)

76 Non-Randomized Controlled Trials as Topic/ (8985)

77 (nonrandom* or non-random* or quasi-random* or quasi-experiment*).tw. (99976)

78 (nRCT or nRCTs or non-RCT$1).tw. (1370)

79 Controlled Before-After Studies/ (195091)

80 (control* adj3 ("before and after" or "before after")).tw. (8006)

81 Interrupted Time Series Analysis/ (186998)

82 (time series adj3 interrupt*).tw. (4055)

83 (pre- adj3 post-).tw. (167334)

84 (pretest adj3 posttest).tw. (8922)

85 Historically Controlled Study/ (205388)

86 (control* adj2 stud$3).tw. (453442)

87 Control Groups/ (111297)

88 (control* adj2 group$1).tw. (994879)

89 trial.ti. (412530)

90 or/73-89 (2788602)

91 46 and 90 [NON-RCTS] (106)

92 exp Cohort Studies/ (2003621)

93 cohort$1.tw. (1102369)

94 Retrospective Studies/ (971861)

95 (longitudinal or prospective or retrospective).tw. (2451448)

96 ((followup or follow-up) adj (study or studies)).tw. (102223)

97 Observational study.pt. (38265)

98 (observation$2 adj (study or studies)).tw. (188524)

99 ((population or population-based) adj (study or studies or analys#s)).tw. (33383)

100 ((multidimensional or multi-dimensional) adj (study or studies)).tw. (222)

101 Comparative Study.pt. (1816271)

102 ((comparative or comparison) adj (study or studies)).tw. (212071)

103 exp Case-Control Studies/ (1015128)

104 ((case-control* or case-based or case-comparison) adj (study or studies)).tw. (198097)

105 or/92-104 (6308345)

106 46 and 105 [OBSERVATIONAL STUDIES] (370)

107 65 or 72 or 91 or 106 [ALL STUDY DESIGNS] (1266)

108 107 use ppez [MEDLINE RECORDS] (353)

109 exp non small cell lung cancer/ (94583)

110 non-small cell lung cancer*.tw,kw. (110361)

111 non-small cell lung carcinoma*.tw,kw. (10443)

112 nonsmall cell lung cancer*.tw,kw. (5615)

113 nonsmall cell lung carcinoma*.tw,kw. (997)

114 ((non-small cell cancer* or nonsmall cell cancer* or non-small cell carcinoma* or nonsmall cell carcinoma*) adj3 lung?).tw,kw. (1272)

115 NSCLC?.tw,kw. (88209)

116 non-small cell bronch* cancer*.tw,kw. (187)

117 non-small cell bronch* carcinoma*.tw,kw. (678)

118 nonsmall cell bronch* cancer*.tw,kw. (5)

119 nonsmall cell bronch* carcinoma*.tw,kw. (18)

120 ((non-small cell cancer* or nonsmall cell cancer* or non-small cell carcinoma* or nonsmall cell carcinoma*) adj3 bronch*).tw,kw. (63)

121 non-small cell pulmonary cancer*.tw,kw. (9)

122 non-small cell pulmonary carcinoma*.tw,kw. (34)

123 nonsmall cell pulmonary cancer*.tw,kw. (4)

124 nonsmall cell pulmonary carcinoma*.tw,kw. (0)

125 ((non-small cell cancer* or nonsmall cell cancer* or non-small cell carcinoma* or nonsmall cell carcinoma*) adj3 pulmonar*).tw,kw. (76)

126 ((lung? or bronch* or pulmonar*) adj1 (adenocarcinoma* or adeno-carcinoma* or adenocancer* or adeno-cancer*)).tw,kw. (31844)

127 large cell lung cancer*.tw,kw. (495)

128 large cell lung carcinoma*.tw,kw. (410)

129 ((large cell cancer* or large cell carcinoma*) adj3 (lung? or bronch* or pulmonar*)).tw,kw. (976)

130 (large cell neuroendocrine cancer* and (lung? or bronch* or pulmonar*)).tw,kw. (28)

131 (large cell neuroendocrine carcinoma* and (lung? or bronch* or pulmonar*)).tw,kw. (1566)

132 ((squamous cell cancer* or squamous cell carcinoma*) and (lung? or bronch* or pulmonar*)).tw,kw. (31839)

133 ((epidermoid cancer* or epidermoid carcinoma* or epidermoid cell cancer* or epidermoid cell carcinoma*) and (lung? or bronch* or pulmonar*)).tw,kw. (2001)

134 ((prickle cell adj (cancer* or carcinoma* or adenocarcinoma* or adeno-carcinoma* or adenocancer* or adeno-cancer*)) and (lung? or bronch* or pulmonar*)).tw,kw. (0)

135 (adenosquamous carcinoma* and (lung? or bronch* or pulmonar*)).tw,kw. (1284)

136 (sarcomatoid carcinoma* and (lung? or bronch* or pulmonar*)).tw,kw. (745)

137 (planocellular carcinoma* and (lung? or bronch* or pulmonar*)).tw,kw. (30)

138 or/109-137 [NSCLC] (202975)

139 exp anaplastic lymphoma kinase inhibitor/ (5405)

140 (anaplastic lymphoma kinase adj3 inhibit*).tw,kw. (644)

141 (ALK adj3 inhibit*).tw,kw. (3399)

142 (ALKI or ALKIs).tw,kw. (52)

143 (crizotinib or "pf 02341066" or pf 1066 or pf 2341066 or pf02341066 or pf1066 or pf2341066 or xalkori).tw,kw,rn. (6919)

144 (ceritinib or ldk 378 or ldk378 or nvp ldk 378 or nvp ldk 378 nx or nvp ldk378 or nvp ldk378 nx or zykadia).tw,kw,rn. (1195)

145 (alectinib or af 802 or af802 or alecensa or alectinib hydrochloride or ch 5424802 or ch5424802 or rg 7853 or rg7853 or ro 5424802 or ro5424802).tw,kw,rn. (951)

146 (brigatinib or ap 26113 or ap26113).tw,kw,rn. (375)

147 (lorlaninib or "pf 06463922" or pf06463922).tw,kw,rn. (142)

148 (ASP3026 or ASP 3026 or UNII-HP4L6MXF10 or 1097917-15-1).tw,kw,rn. (125)

149 (X-396 or X396 or UNII-7DR7JMB8BH or 1365267-27-1).tw,kw,rn. (115)

150 (ensartinib or UNII-SMA5ZS5B22 or 370651-20-9).tw,kw,rn. (10)

151 (entrectinib or nms e 628 or nms e628 or rxdx 101 or rxdx101).tw,kw,rn. (170)

152 or/139-151 [ALK INHIBITORS] (8762)

153 138 and 152 [NSCLC - ALK INHIBITORS] (5139)

154 exp animal experimentation/ or exp animal model/ or exp animal experiment/ or nonhuman/ or exp vertebrate/ (45172606)

155 exp human/ or exp human experimentation/ or exp human experiment/ (35576770)

156 154 not 155 (9597508)

157 153 not 156 [ANIMAL-ONLY REMOVED] (5063)

158 meta-analysis/ (211230)

159 "systematic review"/ (141978)

160 "meta analysis (topic)"/ (35263)

161 (meta-analy* or metanaly* or metaanaly* or met analy* or integrative research or integrative review* or integrative overview* or research integration or research overview* or collaborative review*).tw. (271643)

162 (systematic review* or systematic overview* or evidence-based review* or evidence-based overview* or (evidence adj3 (review* or overview*)) or meta-review* or meta-overview* or meta-synthes* or "review of reviews" or technology assessment* or HTA or HTAs).tw. (317948)

163 biomedical technology assessment/ (21173)

164 (cochrane or health technology assessment or evidence report).jw. (33279)

165 (network adj (MA or MAs)).tw,kw. (14)

166 (NMA or NMAs).tw,kw. (3561)

167 indirect comparison?.tw,kw. (3587)

168 (indirect treatment* adj1 comparison?).tw,kw. (463)

169 (mixed treatment* adj1 comparison?).tw,kw. (1140)

170 (multiple treatment* adj1 comparison?).tw,kw. (198)

171 (multi-treatment* adj1 comparison?).tw,kw. (3)

172 simultaneous comparison?.tw,kw. (863)

173 mixed comparison?.tw,kw. (34)

174 or/158-173 (621639)

175 157 and 174 [SYSTEMATIC REVIEWS] (151)

176 randomized controlled trial/ or controlled clinical trial/ (1177490)

177 exp "clinical trial (topic)"/ (242083)

178 (randomi#ed or randomi#ation or randomly or RCT$1 or placebo*).tw. (1931897)

179 ((singl* or doubl* or trebl* or tripl*) adj (mask* or blind* or dumm*)).tw. (361131)

180 trial.ti. (412530)

181 or/176-180 (2638294)

182 157 and 181 [RCTS] (1104)

183 exp controlled clinical trial/ (1177666)

184 exp "controlled clinical trial (topic)"/ (134916)

185 (control* adj2 trial*).tw. (481900)

186 (nonrandom* or non-random* or quasi-random* or quasi-experiment*).tw. (99976)

187 (nRCT or nRCTs or non-RCT$1).tw. (1370)

188 (control* adj3 ("before and after" or "before after")).tw. (8006)

189 time series analysis/ (19594)

190 (time series adj3 interrupt*).tw. (4055)

191 pretest posttest control group design/ (306)

192 (pre- adj3 post-).tw. (167334)

193 (pretest adj3 posttest).tw. (8922)

194 controlled study/ (5558924)

195 (control* adj2 stud$3).tw. (453442)

196 control group/ (111297)

197 (control* adj2 group$1).tw. (994879)

198 trial.ti. (412530)

199 or/183-198 (7622519)

200 157 and 199 [NON-RCTS] (900)

201 cohort analysis/ (517876)

202 cohort$1.tw. (1102369)

203 retrospective study/ (1208486)

204 longitudinal study/ (212641)

205 prospective study/ (849135)

206 (longitudinal or prospective or retrospective).tw. (2451448)

207 follow up/ (1178540)

208 ((followup or follow-up) adj (study or studies)).tw. (102223)

209 observational study/ (157577)

210 (observation$2 adj (study or studies)).tw. (188524)

211 population research/ (85033)

212 ((population or population-based) adj (study or studies or analys#s)).tw. (33383)

213 ((multidimensional or multi-dimensional) adj (study or studies)).tw. (222)

214 exp comparative study/ (3025743)

215 ((comparative or comparison) adj (study or studies)).tw. (212071)

216 exp case control study/ (1015128)

217 ((case-control* or case-based or case-comparison) adj (study or studies)).tw. (198097)

218 or/201-217 (7948040)

219 157 and 218 [OBSERVATIONAL STUDIES] (1044)

220 175 or 182 or 200 or 219 [ALL RELEVANT STUDY DESIGNS] (2154)

221 220 use oemezd [EMBASE RECORDS] (1888)

222 65 use ppez [MEDLINE REVIEWS] (54)

223 175 use oemezd [EMBASE REVIEWS] (125)

224 222 or 223 (179)

225 remove duplicates from 224 (154)

226 225 use ppez [MEDLINE UNIQUE REVIEWS] (49)

227 225 use oemezd [EMBASE UNIQUE REVIEWS] (105)

228 72 use ppez [MEDLINE RCTS] (142)

229 182 use oemezd [EMBASE RCTS] (1029)

230 228 or 229 (1171)

231 remove duplicates from 230 [TOTAL UNIQUE RCTS] (1050)

232 231 use ppez [MEDLINE UNIQUE RCTS] (126)

233 231 use oemezd [EMBASE UNIQUE RCTS] (924)

234 91 use ppez [MEDLINE NON-RCTS] (29)

235 200 use oemezd [EMBASE NON-RCTS] (860)

236 234 or 235 (889)

237 remove duplicates from 236 [TOTAL UNIQUE NON-RCTS] (832)

238 237 use ppez [MEDLINE UNIQUE NON-RCTS] (24)

239 237 use oemezd [EMBASE UNIQUE NON-RCTS] (808)

240 106 use ppez [MEDLINE OBSERVATIONAL STUDIES] (204)

241 219 use oemezd [EMBASE OBSERVATIONAL STUDIES] (852)

242 240 or 241 (1056)

243 remove duplicates from 242 [TOTAL UNIQUE OBSERVATIONAL STUDIES] (878)

244 243 use ppez [MEDLINE UNIQUE OBSERVATIONAL STUDIES] (180)

245 243 use oemezd [EMBASE UNIQUE OBSERVATIONAL STUDIES] (698)

246 108 or 221 [BOTH DATABASES - ALL STUDY DESIGNS] (2241)

247 remove duplicates from 246 [TOTAL UNIQUE RECORDS - BOTH DATABASES] (1917)

# Appendix 3. Included studies

Note: The list of excluded studies is available from the corresponding author on request.

**Included RCTs** (unique studies and companion reports)

1. Zhou C, Kim SW, Reungwetwattana T, Zhou J, Zhang Y, He J, et al. Alectinib versus crizotinib in untreated Asian patients with anaplastic lymphoma kinase-positive non-small-cell lung cancer (ALESIA): a randomised phase 3 study. Lancet Respir Med. 2019;7(5):437-46.

2. Wilner KD, Usari T, Polli A, Kim EE. Comparison of cardiovascular effects of crizotinib and chemotherapy in ALK-positive advanced non-small-cell lung cancer. Future Oncology. 2019;15(10):1097-103.

3. Li J, Knoll S, Bocharova I, Tang W, Signorovitch J. Comparative efficacy of first-line ceritinib and crizotinib in advanced or metastatic anaplastic lymphoma kinase-positive non-small cell lung cancer: an adjusted indirect comparison with external controls. Curr Med Res Opin. 2019;35(1):105-11.

4. Kawata AK, Lenderking WR, Eseyin OR, Kerstein D, Huang J, Huang H, et al. Converting EORTC QLQ-C30 scores to utility scores in the brigatinib ALTA study. J Med Econ. 2019:1-12.

5. Cho BC, Obermannova R, Bearz A, McKeage M, Kim DW, Batra U, et al. Efficacy and Safety of Ceritinib (450 mg/d or 600 mg/d) With Food Versus 750-mg/d Fasted in Patients With ALK Receptor Tyrosine Kinase (ALK)-Positive NSCLC: Primary Efficacy Results From the ASCEND-8 Study. J Thorac Oncol. 2019;14(7):1255-65.

6. Camidge DR, Dziadziuszko R, Peters S, Mok T, Noe J, Nowicka M, et al. Updated Efficacy and Safety Data and Impact of the EML4-ALK Fusion Variant on the Efficacy of Alectinib in Untreated ALK-Positive Advanced Non-Small Cell Lung Cancer in the Global Phase III ALEX Study. J Thorac Oncol. 2019;14(7):1233-43.

7. Wu YL, Lu S, Lu Y, Zhou J, Shi YK, Sriuranpong V, et al. Results of PROFILE 1029, a Phase III Comparison of First-Line Crizotinib versus Chemotherapy in East Asian Patients with ALK-Positive Advanced Non-Small Cell Lung Cancer. J Thorac Oncol. 2018;13(10):1539-48.

8. Soria JC, Ho SN, Varella-Garcia M, Iafrate AJ, Solomon BJ, Shaw AT, et al. Correlation of extent of ALK FISH positivity and crizotinib efficacy in three prospective studies of ALK-positive patients with non-small-cell lung cancer. Ann Oncol. 2018;29(9):1964-71.

9. Solomon BJ, Kim DW, Wu YL, Nakagawa K, Mekhail T, Felip E, et al. Final Overall Survival Analysis From a Study Comparing First-Line Crizotinib Versus Chemotherapy in ALK-Mutation-Positive Non-Small-Cell Lung Cancer. J Clin Oncol. 2018;36(22):2251-8.

10. Singhi EK, Horn L. Background and rationale of the eXalt3 trial investigating X-396 in the treatment of ALK+ non-small-cell lung cancer. Fut Oncol. 2018;14(18):1781-7.

11. Novello S, Mazieres J, Oh IJ, Castro JD, Migliorino MR, Helland A, et al. Alectinib versus chemotherapy in crizotinib-pretreated anaplastic lymphoma kinase (ALK)-positive non-small-cell lung cancer: results from the phase III ALUR study. Ann Oncol. 2018;29(6):1409-16.

12. Nishio M, Nakagawa K, Mitsudomi T, Yamamoto N, Tanaka T, Kuriki H, et al. Analysis of central nervous system efficacy in the J-ALEX study of alectinib versus crizotinib in ALK-positive non-small-cell lung cancer. Lung Cancer. 2018;121:37-40.

13. Nishio M, Kim DW, Wu YL, Nakagawa K, Solomon BJ, Shaw AT, et al. Crizotinib versus Chemotherapy in Asian Patients with ALK-Positive Advanced Non-small Cell Lung Cancer. Cancer ResTreat. 2018;50(3):691-700.

14. Kiura K, Imamura F, Kagamu H, Matsumoto S, Hida T, Nakagawa K, et al. Phase 3 study of ceritinib vs chemotherapy in ALK-rearranged NSCLC patients previously treated with chemotherapy and crizotinib (ASCEND-5): Japanese subset. Jpn J Clin Oncol. 2018;48(4):367-75.

15. Gadgeel S, Peters S, Mok T, Shaw AT, Kim DW, Ou SI, et al. Alectinib versus crizotinib in treatment-naive anaplastic lymphoma kinase-positive (ALK+) non-small-cell lung cancer: CNS efficacy results from the ALEX study. Ann Oncol. 2018;29(11):2214-22.

16. Camidge DR, Kim HR, Ahn MJ, Yang JC, Han JY, Lee JS, et al. Brigatinib versus Crizotinib in ALK-Positive Non-Small-Cell Lung Cancer. New England Journal of Medicine. 2018;379(21):2027-39.

17. Zhang L, Wu N, Li M, Ying J, Ouyang H, Yang C, et al. The diagnostic and follow-up role of radiological examination in advanced lung adenocarcinoma with anaplastic lymphoma kinase gene rearrangement. International Journal of Clinical and Experimental Medicine. 2017;10(8):12782-9.

18. Yoneda KY, Scranton JR, Cadogan MA, Tassell V, Nadanaciva S, Wilner KD, et al. Interstitial Lung Disease Associated With Crizotinib in Patients With Advanced Non-Small Cell Lung Cancer: Independent Review of Four PROFILE Trials. Clinical Lung Cancer. 2017;(no.

19. Thorne-Nuzzo T, Williams C, Catallini A, Clements J, Singh S, Amberson J, et al. A Sensitive ALK Immunohistochemistry Companion Diagnostic Test Identifies Patients Eligible for Treatment with Crizotinib. Journal of thoracic oncology. 2017;12:804-13.

20. Tan W, Yamazaki S, Johnson TR, Wang R, Gorman MTO, Kirkovsky L, et al. Effects of Renal Function on Crizotinib Pharmacokinetics: Dose Recommendations for Patients with ALK-Positive Non-Small Cell Lung Cancer. Clin Drug Invest. 2017;37:363-73.

21. Soria JC, Tan DS, Chiari R, Wu YL, Paz-Ares L, Wolf J, et al. First-line ceritinib versus platinum-based chemotherapy in advanced ALK-rearranged non-small-cell lung cancer (ASCEND-4): a randomised, open-label, phase 3 study. Lancet. 2017;389:917-29.

22. Shaw AT, Kim TM, Crino L, Gridelli C, Kiura K, Liu G, et al. Ceritinib versus chemotherapy in patients with ALK-rearranged non-small-cell lung cancer previously given chemotherapy and crizotinib (ASCEND-5): a randomised, controlled, open-label, phase 3 trial. Lancet Oncology. 2017.

23. Pfizer. A Study Of Crizotinib Versus Chemotherapy In Previously Untreated ALK Positive East Asian Non-Small Cell Lung Cancer Patients. 2017:NCT01639001.

24. Peters S, Camidge DR, Shaw AT, Gadgeel S, Ahn JS, Kim DW, et al. Alectinib versus crizotinib in untreated ALK-positive non-small-cell lung cancer. The New England journal of medicine. 2017;377(9):828-38. doi: 10.1056/NEJMoa1704795.

25. Kim DW, Tiseo M, Ahn MJ, Reckamp KL, Hansen KH, Kim SW, et al. Brigatinib in patients with crizotinib-refractory anaplastic lymphoma kinase-positive non-small-cell lung cancer: a randomized, multicenter phase II trial. J Clin Oncol. 2017;JCO2016715904.

26. Hida T, Nokihara H, Kondo M, Kim YH, Azuma K, Seto T, et al. Alectinib versus crizotinib in patients with ALK-positive non-small-cell lung cancer (J-ALEX): an open-label, randomised phase 3 trial. Lancet. 2017.

27. Cho BC, Kim DW, Bearz A, Laurie SA, McKeage M, Borra G, et al. ASCEND-8: A Randomized Phase 1 Study of Ceritinib, 450 mg or 600 mg, Taken with a Low-Fat Meal versus 750 mg in Fasted State in Patients with Anaplastic Lymphoma Kinase (ALK)-Rearranged Metastatic Non-Small Cell Lung Cancer (NSCLC). J Thorac Oncol. 2017;12(9):1357-67.

28. Wang E, Nickens DJ, Bello A, Khosravan R, Amantea M, Wilner KD, et al. Clinical implications of the pharmacokinetics of crizotinib in populations of patients with non-small cell lung cancer. Clin Cancer Res. 2016;22:5722-8.

29. Solomon BJ, Cappuzzo F, Felip E, Blackhall FH, Costa DB, Kim DW, et al. Intracranial Efficacy of Crizotinib Versus Chemotherapy in Patients With Advanced ALK-Positive Non-Small-Cell Lung Cancer: Results From PROFILE 1014. J Clin Oncol. 2016;34:2858-65.

30. Ou SH, Tang Y, Polli A, Wilner KD, Schnell P. Factors associated with sinus bradycardia during crizotinib treatment: a retrospective analysis of two large-scale multinational trials (PROFILE 1005 and 1007). Cancer Med. 2016;5:617-22.

31. Hida T, Nakagawa K, Seto T, Satouchi M, Nishio M, Hotta K, et al. Pharmacologic study (JP28927) of alectinib in Japanese patients with ALK+ non-small-cell lung cancer with or without prior crizotinib therapy. Cancer Science. 2016;107:1642-6.

32. Zhao J, Zhang K, Zhang L, Wang H. Clinical Efficacy of Crizotinib in Advanced ALK Positive Non-small Cell Lung Cancer. Zhongguo Fei Ai Za Zhi. 2015;18:616-20.

33. Schnell P, Bartlett CH, Solomon BJ, Tassell V, Shaw AT, Pas TD, et al. Complex renal cysts associated with crizotinib treatment. Cancer Medicine. 2015;4:887-96.

34. Costa DB, Shaw AT, Ou SH, Solomon BJ, Riely GJ, Ahn MJ, et al. Clinical Experience With Crizotinib in Patients With Advanced ALK-Rearranged Non-Small-Cell Lung Cancer and Brain Metastases. J Clin Oncol. 2015;33:1881-8.

35. Solomon BJ, Mok T, Kim DW, Wu YL, Nakagawa K, Mekhail T, et al. First-line crizotinib versus chemotherapy in ALK-positive lung cancer. New England Journal of Medicine. 2014;371:2167-77.

36. Ou SH, Janne PA, Bartlett CH, Tang Y, Kim DW, Otterson GA, et al. Clinical benefit of continuing ALK inhibition with crizotinib beyond initial disease progression in patients with advanced ALK-positive NSCLC. Annals of oncology : official journal of the European Society for Medical Oncology / ESMO. 2014;25:415-22.

37. Lin YT, Wang YF, Yang JCH, Yu CJ, Wu SG, Shih JY, et al. Development of renal cysts after crizotinib treatment in advanced ALK-positive non-small-cell lung cancer. Journal of thoracic oncology. 2014;9:1720-5.

38. Fallet V, Cadranel J, Doubre H, Toper C, Monnet I, Chinet T, et al. Prospective screening for ALK: clinical features and outcome according to ALK status. Eur J Cancer. 2014;50:1239-46.

39. Blackhall F, Kim DW, Besse B, Nokihara H, Han JY, Wilner KD, et al. Patient-reported outcomes and quality of life in PROFILE 1007: a randomized trial of crizotinib compared with chemotherapy in previously treated patients with ALK-positive advanced non-small-cell lung cancer. J Thorac Oncol. 2014;9:1625-33.

40. Shaw AT, Kim DW, Nakagawa K, Seto T, Crino L, Ahn MJ, et al. Crizotinib versus chemotherapy in advanced ALK-positive lung cancer. New England Journal of Medicine. 2013;368:2385-94.

41. Ou SH, Tong WP, Azada M, Siwak-Tapp C, Dy J, Stiber JA. Heart rate decrease during crizotinib treatment and potential correlation to clinical response. Cancer. 2013;119:1969-75.

42. Weickhardt AJ, Scheier B, Burke JM, Gan G, Lu X, Bunn PA, et al. Local ablative therapy of oligoprogressive disease prolongs disease control by tyrosine kinase inhibitors in oncogene-addicted non-small-cell lung cancer. J Thorac Oncol. 2012;7:1807-14.

43. University of C, Spanish Lung Cancer G, Pfizer. EUCROSS: European Trial on Crizotinib in ROS1 Translocated Lung Cancer.

44. Pfizer. "An Investigational Drug, PF-02341066 Is Being Studied Versus Standard Of Care In Patients With Advanced Non-Small Cell Lung Cancer With A Specific Gene Profile Involving The Anaplastic Lymphoma Kinase (ALK) Gene".

45. Novartis P, Novartis. LDK378 Versus Chemotherapy in Previously Untreated Patients With ALK Rearranged Non-small Cell Lung Cancer.

46. Novartis P, Novartis. LDK378 Versus Chemotherapy in ALK Rearranged (ALK Positive) Patients Previously Treated With Chemotherapy (Platinum Doublet) and Crizotinib.

47. Hoffmann-La R. A Study Comparing Alectinib With Crizotinib in Treatment-Naive Anaplastic Lymphoma Kinase-Positive Advanced Non-Small Cell Lung Cancer Participants.

48. Ariad P. "A Study to Evaluate the Efficacy of Brigatinib (AP26113) in Participants With Anaplastic Lymphoma Kinase (ALK)-Positive, Non-small Cell Lung Cancer (NSCLC) Previously Treated With Crizotinib".

# Appendix 4. Risk of bias assessment

Risk of bias was assessed by use of Cochrane’s risk of bias tool for RCTs that reported at least one outcome of interest

**A. Summary of risk of bias across studies**

**B. Risk of bias assessment by domain**

| **Author, year** | **Sequence generation** | **Allocation concealment** | **Blinding, personnel/ participants** | **Blinding, subjective outcomes** | **Blinding, objective outcomes** | **Incomplete outcome data** | **Selective reporting** | **Other threats** |
| --- | --- | --- | --- | --- | --- | --- | --- | --- |
| **Shaw 2013^1^** | Low | Low | High | High | Low | Low | Low | Unclear |
| **Solomon 2014^2^** | Low | Low | High | High | Low | Low | Low | Unclear |
| **Zhao 2015^3^** | Unclear | Unclear | High | High | NA | Low | Unclear | Low |
| **Hida 2016^4^** | Low | Unclear | High | High | NA | Low | Unclear | Unclear |
| **Hida 2017^5^** | Low | Low | High | High | Low | Low | Unclear | Unclear |
| **Kim 2017^6^** | Unclear | Unclear | High | High | Low | Low | Low | Unclear |
| **Peters 2017^7^** | Low | Low | High | High | Low | High | Low | Unclear |
| **Shaw 2017^8^** | Low | Low | High | High | Low | Low | Low | Unclear |
| **Soria 2017^9^** | Low | Low | High | High | Low | Low | Low | Unclear |
| **Camidge 2018^10^** | Unclear | Unclear | High | High | Low | Low | High | Unclear |
| **Novello 2018^11^** | Unclear | Unclear | High | High | Low | Low | Low | Unclear |
| **Wu 2018^12^** | Unclear | Unclear | High | High | Low | Low | Low | Unclear |
| **Zhou 2019^13^** | Low | Low | High | High | Low | Low | High | Unclear |

**C. Detailed risk of bias assessment, by study**

| **Shaw 2013^1^ (PROFILE 1007; NCT00932893)** | | | | | |
| --- | --- | --- | --- | --- | --- |
| **Methods:** Open-label multisite, randomized, phase 3, trial | | | | | |
| **Population:  **18 yr, ALK-positive NSCLC, with ECOG score of 0–2, with progressive disease after one prior platinum-based chemotherapy regimen | | | | | |
| **Interventions:** Crizotinib (250 mg BID) in 3-wk cycles v. pemetrexed (500 mg/m^2^) or docetaxel (75 mg/m^2^) | | | | | |
| **Cross-over between treatment groups:** Not during study period; participants from the chemotherapy arm could enroll in NCT00932451 | | | | | |
| **Reported outcomes of interest to this review:**  • TR death;  • OS;  • PFS (Time in months from randomization to first documentation of objective disease progression as determined by independent radiology review or to death due to any cause, whichever occurred first. Progression is defined using Response Evaluation Criteria in Solid Tumors Criteria version 1.1 (RECIST v1.1), as at least a 20% increase (including an absolute increase of at least 5 mm) in the sum of diameters of target lesions, taking as reference the smallest sum on study and/or unequivocal progression of existing non-target lesions and/or appearance of 1 or more new lesions.  • SAEs (an adverse event that results in death, is life threatening, requires inpatient hospitalization or extends a current hospital stay, results in ongoing or significant incapacity or interferes substantially with normal life functions, or causes a congenital anomaly or birth defect) | | | | | |
| **Risk of bias** | | | | | |
| **Domain** | | **Judgment** | | | **Support for judgment** |
| Random sequence generation | | Low risk | | | Centralized Interactive Voice Response System (IVRS)/website |
| Allocation concealment | | Low risk | | | Centralized allocation |
| Blinding of participants and personnel | | High risk | | | Open label study in which knowledge of group assignment might have influenced performance |
| Blinding of outcome assessment: SUBJECTIVE OUTCOMES | | High risk | | | Open label study in which knowledge of group assignment might have affected subjective outcome assessment, e.g., treatment-related death that involves judgment on the causal relationship between drug and death |
| Blinding of outcome assessment: OBJECTIVE OUTCOMES | | Low risk | | | Despite being open label, knowledge of group assignment would not be expected to affect objective outcomes assessment. A central blinded independent radiologic review was conducted for PFS |
| Incomplete outcome data | | Low risk | | | Lost to follow-up: chemotherapy 2/174 (1%), crizotinib 4/173 (2%); withdrawn consent: chemotherapy 3/174 (2%), crizotinib 2/173 (1%); other reasons or protocol violations: chemotherapy 12/173 (7%), crizotinib 2/173 (1%). Unbalanced in terms of withdrawals due to “other reasons/protocol violations” in the chemotherapy group. However, given the comparatively low numbers and percentage of the whole population, the risk of attrition bias is judged to be low |
| Selective reporting | | Low risk | | | No detected difference in reporting between protocol and publication |
| Other potential threats to validity | | Unclear | | | • Pharmaceutical funding (Pfizer)  • Participants in the chemotherapy group with disease progression were permitted to receive crizotinib as part of an additional study (NCT00932451); however, the authors note that their analysis of overall survival was “likely confounded by the high crossover rate among patients in the chemotherapy group."  • OS data were immature at data analysis; interim analysis performed |
| **Solomon 2014^2^ (PROFILE 1014; NCT01154140)** | | | | | |
| **Methods:** Open-label, multi-center, randomized, phase 3 trial | | | | | |
| **Population:** Patients with advanced, non-squamous ALK-positive NSCLC | | | | | |
| **Interventions:** Crizotinib (250 mg BID) in 21 day cycles v. pemetrexed (500 mg/m^2^) or cisplatin (75 mg/m^2^) for up to six 21-day cycles | | | | | |
| **Cross-over between treatment groups:** Yes; participants in the chemotherapy arm with disease progression could cross to the crizotinib arm provided safety criteria were met | | | | | |
| **Reported outcomes of interest to this review:**  **•** TR death;  • OS;  • PFS (time from the date of randomization in study until the date of first documented objective tumor progression (according to RECIST v1.1 as determined by IRR) or death (due to any cause), whichever occurred first. Objective progression was defined as a 20 percent (%) increase in the sum of the diameters of target measurable lesions taking as reference the smallest sum on study (this includes the baseline sum if that is the smallest on study), with a minimum absolute increase of 5 millimeter (mm) or clear progression of pre-existing non-target lesions, or the appearance of any new clear lesions.)  • SAEs (an adverse event that results in death, is life threatening, requires inpatient hospitalization or extends a current hospital stay, results in ongoing or significant incapacity or interferes substantially with normal life functions, or causes a congenital anomaly or birth defect) | | | | | |
| **Risk of bias** | | | | | |
| **Domain** | | **Judgment** | | | **Support for judgment** |
| Random sequence generation | | Low risk | | | Permuted block randomization via centralized interactive voice response system or website |
| Allocation concealment | | Low risk | | | Centralized allocation |
| Blinding of participants and personnel | | High risk | | | Open label study in which knowledge of group assignment might have influenced performance |
| Blinding of outcome assessment: SUBJECTIVE OUTCOMES | | High risk | | | Open label study in which knowledge of group assignment might have affected subjective outcome assessment, e.g., treatment-related death that involves judgment on the causal relationship between drug and death. |
| Blinding of outcome assessment: OBJECTIVE OUTCOMES | | Low risk | | | Despite being open label, knowledge of group assignment would not be expected to affect objective outcomes assessment. PFS was assessed by independent review. |
| Incomplete outcome data | | Low risk | | | Loss to follow-up: chemotherapy 7/171 (4%), crizotinib 7/172 (4%); withdrew consent: chemotherapy 9/172 (5), crizotinib 3/171 (2%); “other reasons”: chemotherapy 7/171 (4%); crizotinib 1/172 (1%). Other reasons and protocol violations were not described, but few people in each group were withdrawn for this reason. |
| Selective reporting | | Low risk | | | No detected difference in reporting between protocol and publication |
| Other potential threats to validity | | Unclear | | | • Pharmaceutical funding (Pfizer)  • Cross-over between treatment groups was permitted; however, steps were taken to mitigate this potential bias: "As prespecified in the protocol, overall survival was also analyzed with the rank-preserving structural failure time model to explore the effect of crossover to crizotinib in the chemotherapy group. All analyses in the chemotherapy group, with the exception of the analysis of overall survival, included only data collected before crossover to crizotinib." |
| **Zhao 2015^3^ (Chinese-language publication)** | | | | | |
| **Methods:** Randomized controlled trial | | | | | |
| **Population:**  18 yr, ALK-positive NSCLC, Karnofsky performance status (KPS) score ≥ 70, following first- or second-line chemotherapy | | | | | |
| **Interventions:** Crizotinib (250 mg BID) v. chemotherapy docetaxel injection (75 mg/m^2^ every three weeks for at least 3 cycles) | | | | | |
| **Cross-over between treatment groups:** Not reported | | | | | |
| **Reported outcomes of interest to this review:**  **•** TR death | | | | | |
| **Risk of bias** | | | | | |
| **Domain** | **Judgment** | | **Support for judgment** | | |
| Random sequence generation | Unclear | | Insufficient information reported to permit judgment | | |
| Allocation concealment | Unclear | | Insufficient information reported to permit judgment | | |
| Blinding of participants and personnel | High risk | | Blinding was not reported and was likely not done because one treatment was administered orally (crizotinib) and one by injection (chemotherapy). Knowledge of group assignment might have influenced performance | | |
| Blinding of outcome assessment: SUBJECTIVE OUTCOMES | High risk | | Open label study in which knowledge of group assignment might have affected subjective outcome assessment, e.g., treatment-related death that involves judgment on the causal relationship between drug and death. | | |
| Blinding of outcome assessment: OBJECTIVE OUTCOMES | NA | | NA | | |
| Incomplete outcome data | Low risk | | No patients were lost to follow-up | | |
| Selective reporting | Unclear | | No protocol available | | |
| Other potential threats to validity | Low risk | | This study was funded by the grant from National Natural Science Foundation of China. No potential threats to validity were noted. | | |
| **Hida 2016^4^ (JP28927; JapicCTI-132186)** | | | | | |
| **Methods**: Multicenter, open-label randomized, pharmacologic study; 3-period cross-over | | | | | |
| **Population**:  20 yr, ALK-positive NSCLC, with ECOG score of 0–1; Prior treatment, including other ALK inhibitors, was allowed | | | | | |
| **Interventions**: “Patients in group A received alectinib 20/40-mg capsules for 10 days (fasting), followed by 150-mg capsules for 10 days (fasting), then 150-mg capsules for 10 days (nonfasting state). Patients in group B received alectinib 150-mg capsules for 10 days (fasting), followed by 20/40-mg capsules for 10 days (fasting), then 150-mg capsules for 10 days (non-fasting state). After cycle 1, patients received 150-mg alectinib capsules until investigator-determined lack of clinical benefit” | | | | | |
| **Cross-over between treatment groups:** Yes, by design during cross-over phase | | | | | |
| **Reported outcomes of interest to this review:** TR death | | | | | |
| **Risk of bias** | | | | | |
| **Domain** | **Judgment** | | **Support for judgment** | | |
| Random sequence generation | Low risk | | Permuted block randomization method was used; | | |
| Allocation concealment | Unclear | | Insufficient information available for judgment | | |
| Blinding of participants and personnel | High risk | | Open label study in which knowledge of group assignment might have influenced performance | | |
| Blinding of outcome assessment: SUBJECTIVE OUTCOMES | High risk | | Open label study in which knowledge of group assignment might have affected subjective outcome assessment, e.g., treatment-related death that involves judgment on the causal relationship between drug and death. | | |
| Blinding of outcome assessment: OBJECTIVE OUTCOMES | NA | | NA | | |
| Incomplete outcome data | Low risk | | No patients were lost to follow-up | | |
| Selective reporting | Unclear | | A protocol was referred to in the publication but was not available | | |
| Other potential threats to validity | Unclear | | • Pharmaceutical funding (Chugai Pharmaceutical Co.) • Data not reported for phase 1 (crossover between fasting conditions) | | |
| **Hida 2017^5^ (J-ALEX; JAPICcti-132316)** | | | | | |
| **Methods:** Open-label, multi-site, randomized, phase 3 trial (Japan) | | | | | |
| **Participants:**  20 yr, ALK-positive NSCLC, with ECOG score of 0–2, ALK-inhibitor naive, chemotherapy-naïve or had received 1 regimen of chemotherapy | | | | | |
| **Interventions:** Crizotinib (250 mg BID) v. Alectinib (300 mg BID) | | | | | |
| **Cross-over between treatment groups:** No: Treatment crossover after study withdrawal was allowed in both study groups. | | | | | |
| **Reported outcomes of interest to this review:**  **•** TR death;  • PFS (independent review; progressive disease or death according to RECIST version 1.1) | | | | | |
| **Risk of Bias** | | | | | |
| **Domain** | **Judgment** | | **Quote** | | |
| **Bias** | **Judgment** | | **Support for judgment** | | |
| Random sequence generation | Low risk | | Interactive web response system was used for sequence generation | | |
| Allocation concealment | Low risk | | Interactive web response system was used for allocation concealment | | |
| Blinding of participants and personnel | High risk | | Open label study in which knowledge of group assignment might have influenced performance | | |
| Blinding of outcome assessment: SUBJECTIVE OUTCOMES | High risk | | Open label study in which knowledge of group assignment might have affected subjective outcome assessment, e.g., treatment-related death that involves judgment on the causal relationship between drug and death. | | |
| Blinding of outcome assessment: OBJECTIVE OUTCOMES | Low risk | | Despite being open label, knowledge of group assignment would not be expected to affect objective outcomes assessment. Blinded assessment of PFS | | |
| Incomplete outcome data | Low risk | | No patients were lost to follow-up. | | |
| Selective reporting | Unclear | | Protocol not available | | |
| Other potential threats to validity | Unclear | | • Pharmaceutical funding (Chugai Pharmaceutical Co.)  • Unclear description of crossover between groups: "Treatment crossover after study withdrawal was allowed in both study groups. Patients initially randomly assigned to the crizotinib group who withdrew from the study because of progressive disease before the approval of alectinib (ie, before September, 2014), were permitted to receive alectinib during the study."  • OS data were immature | | |
| **Kim 2017^6^ (ALTA, NCT02094573)** | | | | | |
| **Methods:** Open-label, randomized, multicenter phase 2 trial | | | | | |
| **Participants:**  18 yr, ALK-positive NSCLC, with ECOG performance status of 0–2, disease progression while receiving crizotinib | | | | | |
| **Interventions:** Brigatinib 90 mg QD v. Brigatinib 180 mg QD | | | | | |
| **Cross-over between treatment groups:** Yes, participants in the 90 mg/d group could cross to the 180 mg/d group after disease progression | | | | | |
| **Reported outcomes of interest to this review:**  • PFS (independent review committee (IRC), per RECIST v1.1;  time interval from the date of the first dose of the study treatment until the first date at which disease progression is objectively documented, or death due to any cause, whichever occurs first; Disease progression for target lesion: SLD increased by at least 20% from the smallest value on study (including baseline, if that is the smallest) and SLD must also demonstrate an absolute increase of at least 5 mm or development of any new lesion. Disease progression for non-target lesion: Unequivocal progression of existing non-target lesions.)  • SAEs (an adverse event that results in death, is life threatening, requires inpatient hospitalization or extends a current hospital stay, results in ongoing or significant incapacity or interferes substantially with normal life functions, or causes a congenital anomaly or birth defect) | | | | | |
| **Domain** | **Judgment** | | **Quote** | | |
| **Bias** | **Judgment** | | **Support for judgment** | | |
| Random sequence generation | Unclear | | Randomization was mentioned; no methods were reported for sequence generation. | | |
| Allocation concealment | Unclear | | Randomization was mentioned; no methods were reported for allocation concealment. | | |
| Blinding of participants and personnel | High risk | | Open label study in which knowledge of group assignment might have influenced performance | | |
| Blinding of outcome assessment: SUBJECTIVE OUTCOMES | High risk | | Open label study in which knowledge of group assignment might have affected subjective outcome assessment, e.g., treatment-related death that involves judgment on the causal relationship between drug and death. | | |
| Blinding of outcome assessment: OBJECTIVE OUTCOMES | Low risk | | Despite being open label, knowledge of group assignment would not be expected to affect objective outcomes assessment | | |
| Incomplete outcome data | Low risk | | No patients were lost to follow-up | | |
| Selective reporting | Low risk | | Selective reporting not detected | | |
| Other potential threats to validity | Unclear | | • Pharmaceutical funding (ARIAD Pharmaceuticals)  • Ongoing study | | |
| **Peters 2017^7^ (ALEX; NCT02075840)** | | | | | |
| **Methods:** Open-label, multi-site, randomized, phase 3 trial | | | | | |
| **Participants:**  18 yr, ALK-positive NSCLC, with ECOG score of 0–2, with no prior systemic treatment | | | | | |
| **Interventions:** Alectinib (600 mg BID) or crizotinib (250 mg BID) | | | | | |
| **Cross-over between treatment groups:** No: “Per protocol, crossover between trial groups was not allowed; patients assigned to crizotinib may have received alectinib after disease progression (in countries where alectinib was already approved or available).” | | | | | |
| **Reported outcomes of interest to this review:**  • TR death • OS  • PFS (investigator assessed [primary]; independent review;  time to disease progression or death whichever occurred first, by use of Response Evaluation Criteria in Solid Tumors (RECIST) Version 1.1 (v1.1) Criteria; PFS was assessed as time to disease progression or death whichever occurred first by investigator assessment using Response Evaluation Criteria in Solid Tumors (RECIST) Version 1.1 (v1.1) Criteria. As per RECIST v1.1, disease progression is a 20% increase in the sum of the diameters of target lesions, an increase in size of measurable lesions by at least 5 millimeter (mm) and the appearance of new lesions.)  • SAEs (an adverse event that results in death, is life threatening, requires inpatient hospitalization or extends a current hospital stay, results in ongoing or significant incapacity or interferes substantially with normal life functions, or causes a congenital anomaly or birth defect) | | | | | |
| **Risk of bias** | | | | | |
| **Bias** | | **Judgment** | | **Support for judgment** | |
| Random sequence generation | | Low risk | | Participants were randomly allocated by use of an interactive or Web-based system | |
| Allocation concealment | | Low risk | | Allocation was concealed by use of an interactive or Web-based system | |
| Blinding of participants and personnel | | High risk | | Open label study in which knowledge of group assignment might have influenced performance | |
| Blinding of outcome assessment: SUBJECTIVE OUTCOMES | | High risk | | Open label study in which knowledge of group assignment might have affected subjective outcome assessment, e.g., treatment-related death that involves judgment on the causal relationship between drug and death. | |
| Blinding of outcome assessment: OBJECTIVE OUTCOMES | | Low risk | | Despite being open label, knowledge of group assignment would not be expected to affect objective outcomes assessment. The primary outcome (PFS) was based on unblinded investigator’s assessment; secondary analysis using independent assessment; however, the two estimates were similar: (Investigator HR: 0.47 [95% CI, 0.34 to 0.65]; independent review HR: 0.50 [95% CI, 0.36 to 0.70] | |
| Incomplete outcome data | | High risk | | Lost to follow-up or “declined to participate”: alectinib 17/152 (10%), crizotinib 27/151 (18%); discontinued treatment due to “other reasons”: alectinib 3/152 (2%), crizotinib 2/151 (1%); unbalanced discontinuations and withdrawals between groups could have affected the direction and magnitude of the effect size | |
| Selective reporting | | Low risk | | No differences in outcomes between the protocol and publication were detected | |
| Other potential threats to validity | | Unclear risk | | • Interim analysis; OS data immature  • Pharmaceutical funding (Hoffmann–La Roche) | |
| **Shaw 2017^8^ (ASCEND-5, NCT01828112)^8,14^** | | | | | |
| **Methods**: Open-label, multisite, randomized, phase 3 trial | | | | | |
| **Population**:  18 yr, ALK-positive NSCLC, with WHO performance status of 0–2, one or two previous chemotherapy regimens and previous crizotinib for at least 21 d | | | | | |
| **Interventions**: Ceritinib (750 mg per day, fasted, in continuous 21 day treatment cycles) v. chemotherapy (intravenous pemetrexed 500 mg/m² or docetaxel 75 mg/m² [investigator choice], every 21 days). | | | | | |
| **Cross-over between treatment groups:** Yes, participants in the chemotherapy arm could cross over to the ceritinib group after disease progression | | | | | |
| **Reported outcomes of interest to this review:**  **•** TR death  • OS  • PFS (independent review; PFS is defined as the time from the date of randomization to the date of the first radiologically documented disease progression or death due to any cause.)  • SAEs (an adverse event that results in death, is life threatening, requires inpatient hospitalization or extends a current hospital stay, results in ongoing or significant incapacity or interferes substantially with normal life functions, or causes a congenital anomaly or birth defect) | | | | | |
| **Risk of bias** | | | | | |
| **Domain** | | **Judgment** | | **Support for judgment** | |
| Random sequence generation | | Low risk | | Centralized interactive voice response system /website | |
| Allocation concealment | | Low risk | | Centralized allocation | |
| Blinding of participants and personnel | | High risk | | Open label study in which knowledge of group assignment might have affected subjective outcome assessment, e.g., treatment-related death that involves judgment on the causal relationship between drug and death. | |
| Blinding of outcome assessment: SUBJECTIVE OUTCOMES | | High risk | | Open label study in which knowledge of group assignment might have affected subjective outcome assessment, e.g., treatment-related death that involves judgment on the causal relationship between drug and death. | |
| Blinding of outcome assessment: OBJECTIVE OUTCOMES | | Low risk | | Despite being open label, knowledge of group assignment would not be expected to affect objective outcomes assessment. Outcomes were assessed both locally by investigators and by a masked independent review committee | |
| Incomplete outcome data | | Low risk | | No patients were lost to follow-up. Withdrawal due to patient or guardian decision: chemotherapy 7/105 (7%), ceritinib 6/82 (7%). Withdrawal due to physician decision: chemotherapy 3/105 (3%), ceritinib 5/82 (6%). Given the low and balanced discontinuations and withdrawals between groups, attrition bias is estimated to be modest. | |
| Selective reporting | | Low risk | | No detected difference in reporting between protocol and publication | |
| Other potential threats to validity | | Unclear | | • Pharmaceutical funding (Novartis)  • Cross-over to ceritinib permitted during the extension; authors note that differences in overall survival were “probably confounded by the high proportion of cross-over of patients from the chemotherapy group to the ceritinib group”  • Interim analysis; OS data immature | |
| **Soria 2017^9^ (ASCEND-4; NCT01828099)** | | | | | |
| **Methods:** Open-label, randomized, multi-national, phase 3 study | | | | | |
| **Population:**  18 yr, ALK-positive NSCLC, ECOG score of 0–2, previously untreated | | | | | |
| **Interventions:** Ceritinib 750 mg/d v. intravenous chemotherapy (cisplatin [75 mg/m²], or carboplatin [target area under the curve of 5–6] plus pemetrexed [500 mg/m²]) given every 21 days | | | | | |
| **Cross-over between treatment groups**: Yes, participants in the chemotherapy arm could crossover to ceritinib after disease progression | | | | | |
| **Reported outcomes of interest to this review:**  **•** TR death  • OS  • PFS (PFS defined as time from date of randomization to date of first documented disease (as assessed by Blinded Independent Review Committee (BIRC) per RECIST 1.1) or date of death due to any cause)  • SAEs (an adverse event that results in death, is life threatening, requires inpatient hospitalization or extends a current hospital stay, results in ongoing or significant incapacity or interferes substantially with normal life functions, or causes a congenital anomaly or birth defect) | | | | | |
| **Bias** | | **Judgment** | | **Support for judgment** | |
| Random sequence generation | | Low risk | | Patients were randomized via use of “Interactive Response Technology” (includes Interactive Voice Response System and Interactive Web Response System) | |
| Allocation concealment | | Low risk | | Centralized allocation | |
| Blinding of participants and personnel | | High risk | | Open label study in which knowledge of group assignment might have influenced performance | |
| Blinding of outcome assessment: SUBJECTIVE OUTCOMES | | High risk | | Open label study in which knowledge of group assignment might have affected subjective outcome assessment, e.g., treatment-related death that involves judgment on the causal relationship between drug and death. | |
| Blinding of outcome assessment: OBJECTIVE OUTCOMES | | Low risk | | Despite being open label, knowledge of group assignment would not be expected to affect objective outcomes assessment | |
| Incomplete outcome data | | Low risk | | Withdrawals due to “other” reasons: chemotherapy 9/187 (5%), ceritinib 12/189 (6%)  Given the low and balanced discontinuations and withdrawals between groups, the risk of attrition bias is estimated to be modest. | |
| Selective reporting | | Low risk | | No detected difference in reporting between protocol and publication | |
| Other potential threats to validity | | Unclear | | • Pharmaceutical funding (Novartis)  • Cross-over between treatment groups allowed  • Interim analysis | |
| **Camidge 2018^10^ (ALTA-1L; NCT02737501)** | | | | | |
| **Methods:** Open-label, randomized, multi-national, phase 3 study | | | | | |
| **Population:**  18 yr, ALK-positive locally advanced or metastatic NSCLC with at least one measurable lesion according to the Response Evaluation Criteria in Solid Tumors (RECIST), version 1; had not previously received an ALK-targeted therapy | | | | | |
| **Interventions:** Oral brigatinib (180 mg once daily after a 7-day lead-in period of 90 mg once daily) v. oral crizotinib at a dose of 250 mg twice daily | | | | | |
| **Cross-over between treatment groups:** Yes: “In the crizotinib group, crossover to brigatinib was permitted after progression assessed by means of blinded independent review(with a 10-day washout period from crizotinib)” | | | | | |
| **Reported outcomes of interest to this review:**  **•** TR death  • OS  • PFS (PFS as assessed by blinded Independent Review Committee (BIRC) is defined as the time interval from the date of randomization until the first date at which disease progression (PD) is objectively documented, or death due to any cause, whichever occurs first. PD is sum of longest diameter (SLD) increased by at least 20 percent (%) from the smallest value on study (including baseline, if that is the smallest), the SLD must also demonstrate an absolute increase of at least 5 millimeter (mm), and unequivocal progression of existing non-target lesions.) | | | | | |
| **Risk of bias** | | | | | |
| **Domain** | | **Judgment** | | **Support for judgment** | |
| Random sequence generation | | Unclear | | Randomization procedure not described | |
| Allocation concealment | | Unclear | | Allocation concealment not described | |
| Blinding of participants and personnel | | High risk | | Open label study in which knowledge of group assignment might have influenced performance | |
| Blinding of outcome assessment: SUBJECTIVE OUTCOMES | | High risk | | Open label study in which knowledge of group assignment might have affected subjective outcome assessment, e.g., treatment-related death that involves judgment on the causal relationship between drug and death. | |
| Blinding of outcome assessment: OBJECTIVE OUTCOMES | | Low risk | | Despite being open label, knowledge of group assignment would not be expected to affect objective outcomes assessment | |
| Incomplete outcome data | | Low risk | | More discontinuations occurred in the crizotinib group mainly because of disease progression; about half crossed over to the other arm; however, PFS was assessed before cross-over; interim analysis was appropriate with alpha spending functions. | |
| Selective reporting | | High risk | | Published protocol includes a section titled "exploratory endpoints" but the information has been redacted | |
| Other potential threats to validity | | Unclear | | • Pharmaceutical funding (Ariad Pharmaceuticals) • Cross-over between treatment groups allowed  • Interim analysis | |
| **Novello 2018^11^ (ALUR; NCT02604342)** | | | | | |
| **Methods:** Open-label, randomized, multi-national, phase 3 study | | | | | |
| **Population:** histologically/cytologically confirmed advanced, recurrent, or metastatic ALK-positive NSCLC; two prior lines of systemic therapy (including one line of PDC and one of crizotinib); measurable disease (Response Evaluation Criteria in Solid Tumors [RECIST] v1.1); Eastern  Cooperative Oncology Group performance status (ECOG PS) 0–2. | | | | | |
| **Interventions:** Alectinib 600 mg twice daily v. chemotherapy (pemetrexed 500 mg/m2 or docetaxel 75 mg/m2, every 3 weeks, at the  investigators’ discretion) | | | | | |
| **Cross-over between treatment groups:** Yes: “Crossover from chemotherapy to alectinib was permitted following  progression.” | | | | | |
| **Reported outcomes of interest to this review:**  • OS  • PFS (investigator-assessed [primary]; secondary analysis included independent assessment; time from randomization to the first documented disease progression, as determined using RECIST v1.1, or death from any cause, whichever occurred first. As per RECIST v1.1, disease progression is a 20% increase in the sum of the diameters of target lesions, an increase in size of measurable lesions by at least 5 millimeter (mm) and the appearance of new lesions)  • SAEs (an adverse event that results in death, is life threatening, requires inpatient hospitalization or extends a current hospital stay, results in ongoing or significant incapacity or interferes substantially with normal life functions, or causes a congenital anomaly or birth defect) | | | | | |
| **Risk of bias** | | | | | |
| **Domain** | | **Judgment** | | **Support for judgment** | |
| Random sequence generation | | Unclear | | Randomization procedure not described | |
| Allocation concealment | | Unclear | | Allocation concealment not described | |
| Blinding of participants and personnel | | High risk | | Open label study in which knowledge of group assignment might have influenced performance | |
| Blinding of outcome assessment: SUBJECTIVE OUTCOMES | | High risk | | Open label study in which knowledge of group assignment might have affected subjective outcome assessment; Primary outcome (PFS) was assessed by unblinded investigators, while independently reviewed PFS was provided in secondary analyses. Effect estimates were different between two assessments (Investigator assessed: HR 0.16, 95% CI: 0.09–0.30; Independent review: HR 0.32, 95% CI: 0.17–0.59) | |
| Blinding of outcome assessment: OBJECTIVE OUTCOMES | | Low risk | | Despite being open label, knowledge of group assignment would not be expected to affect objective outcomes assessment. | |
| Incomplete outcome data | | Low risk | | The ITT population comprised all patients randomized. | |
| Selective reporting | | Low risk | | Data for outcomes listed in NCT record have been reported in the full publication or in the NCT record | |
| Other potential threats to validity | | Unclear | | • Pharmaceutical funding (Hoffmann-La Roche) • Cross-over between treatment groups allowed  • Overall survival data immature at cut-off | |
| **Wu 2018^12^ (NCT01639001)** | | | | | |
| Methods: Open-label, randomized, multi-national, phase 3 | | | | | |
| Population: Aged 18-70 years, ALK-positive NSCLC, with no prior systemic treatment, measurable disease according to Response Evaluation criteria in Solid Tumors (RECIST) version 1.1; ECOG PS 0 to 2; adequate hepatic, renal and bone marrow function | | | | | |
| Interventions: Crizotinib 250 mg BID v. Pemetrexed (500 mg/m^2^)/Cisplatin (75 mg/m^^2^)Or Pemetrexed(500 mg/m^2^)/Carboplatin (AUC of 5 or 6 mg.min/mL) | | | | | |
| Cross-over between treatment groups: Not reported | | | | | |
| Reported outcomes of interest to this review:  • TR death  • OS  • PFS (independent review; time from the date of randomization to the date of the first documentation of objective tumor progression (by IRR) or death on study due to any cause, whichever occured first. If tumor progression data included more than 1 date, the first date was used. PFS (in months) was calculated as (first event date − randomization date +1)/30.44. Progression is defined using RECIST v1.1, as at least a 20% increase (including an absolute increase of at least 5 millimeters) in the sum of diameters of target lesions, taking as reference the smallest sum on study and/or unequivocal progression of existing non-target lesions and/or appearance of 1 or more new lesions)  • SAEs (an adverse event that results in death, is life threatening, requires inpatient hospitalization or extends a current hospital stay, results in ongoing or significant incapacity or interferes substantially with normal life functions, or causes a congenital anomaly or birth defect) | | | | | |
| **Risk of bias** | | | | | |
| **Bias** | | **Judgment** | | **Support for judgment** | |
| Random sequence generation | | Unclear | | Insufficient details to permit judgment | |
| Allocation concealment | | Unclear | | Insufficient details to permit judgment | |
| Blinding of participants and personnel | | High risk | | Open label study in which knowledge of group assignment might have affected subjective outcome assessment, e.g., treatment-related death that involves judgment on the causal relationship between drug and death. | |
| Blinding of outcome assessment: SUBJECTIVE OUTCOMES | | High risk | | Open label study in which knowledge of group assignment might have affected subjective outcome assessment, e.g., treatment-related death that involves judgment on the causal relationship between drug and death. | |
| Blinding of outcome assessment: OBJECTIVE OUTCOMES | | Low risk | | Despite being open label, knowledge of group assignment would not be expected to affect objective outcome assessment; PFS assessed by independent review | |
| Incomplete outcome data | | Low risk | | One patient was lost-to follow-up in the crizotinib group (of 104), with none lost in the chemotherapy group (of 103); 3 patients in the crizotinib group refused further follow-up, compared with 6 in the chemotherapy group. | |
| Selective reporting | | Low risk | | No detected selective reporting | |
| Other potential threats to validity | | Unclear | | • Pharmaceutical funding (Pfizer) • Ongoing study (data provided up to 2015 cut point)  • 80.1% of patients from chemotherapy arm crossed over to the crizotinib arm | |
| **Zhou 2019^13^ (ALESIA; NCT02838420)** | | | | | |
| **Methods:** Open-label, randomized, multi-national, phase 3 study | | | | | |
| **Population:**  18 yr Asian patients with histologically or cytologically confirmed stage 3b or 4 ALK-positive NSCLC, had not received previous systemic therapy for advanced NSCLC, had measurable disease at baseline (according to Response Evaluation Criteria in Solid Tumours [RECIST] version 1.1), an Eastern Cooperative Oncology Group performance status (ECOG PS) of 0–2, and a life expectancy of at least 12 weeks. | | | | | |
| **Interventions:** Alectinib (600 mg twice per day; oral) v. crizotinib (250 mg twice per day; oral with or without food) | | | | | |
| **Cross-over between treatment groups:** No: “Crossover between study groups was not permitted” | | | | | |
| **Reported outcomes of interest to this review:**  **•** TR death  • OS  • PFS (investigator assessed; time (in months) from randomization to the first documentation of disease progression, as determined by the investigators, or to death from any cause, whichever occurred first; Determined by Investigator Using Response Evaluation Criteria in Solid Tumor (RECIST) v1.1)  • SAEs (an adverse event that results in death, is life threatening, requires inpatient hospitalization or extends a current hospital stay, results in ongoing or significant incapacity or interferes substantially with normal life functions, or causes a congenital anomaly or birth defect) | | | | | |
| **Risk of bias** | | | | | |
| **Domain** | | **Judgment** | | **Support for judgment** | |
| Random sequence generation | | Low risk | | "Randomisation was done centrally via an interactive voice or web response system" "Patients were randomly assigned via a block-stratified (block size three) randomisation procedure in a 2:1 ratio…" | |
| Allocation concealment | | Low risk | | Randomization was done centrally via an interactive voice or web response system | |
| Blinding of participants and personnel | | High risk | | "Clinical staff involved in the study at investigative sites and the funder’s drug safety and medical monitoring staff had access to information outlining the treatments assigned to individual patients during the study to monitor safety and to do routine data cleaning activities. " | |
| Blinding of outcome assessment: SUBJECTIVE OUTCOMES | | High risk | | Outcome assessors were not blinded to treatment arm; Primary outcome (PFS) was assessed by unblinded investigators; Secondary endpoints included independent review committee-assessed PFS; differences in effect estimate for investigator assessment (HR 0.22, 95% CI 0.13–0.38) and independent assessment (HR 0.37, 95% CI 0.22–0.61) were noted | |
| Blinding of outcome assessment: OBJECTIVE OUTCOMES | | Low risk | | Despite being open label, knowledge of group assignment would not be expected to affect objective outcomes assessment. | |
| Incomplete outcome data | | Low risk | | Discontinuations similar across groups for the discontinued treatment due to withdrew consent (1 vs. 1); primary analysis based on ITT; no cross-over allowed | |
| Selective reporting | | High risk | | No protocol available; NCT record lists 12 outcomes and but data not reported for all outcomes (not stated why data reported for some but not all outcomes) | |
| Other potential threats to validity | | Unclear | | • Pharmaceutical funding (Hoffmann-La Roche): “The funder was involved in the study design, data collection, data analysis, data interpretation, and writing of the Article.”  • Overall survival data immature at cut-off | |
| Note: OS = overall survival, PFS = progression-free survival | | | | | |

# Appendix 5: Model diagnostics

**A. Model diagnosis statistics: Meta-analyses**

| **Outcome** | **Fixed Effects** | | **Random Effects** | |
| --- | --- | --- | --- | --- |
|  | **DIC** | **Residual deviance** | **DIC** | **Residual deviance** |
| **Treatment-related deaths** |  |  |  |  |
| All patients | 36.60 | 7.11 vs 12 data points | 37.12 | 7.31 vs 12 data points |
| Naïve | 36.60 | 7.11 vs 12 data points | 37.12 | 7.31 vs 12 data points |
| Experienced | 19.13 | 4.16 vs 6 data points | 19.22 | 4.19 vs 6 data points |
| **Overall survival** |  |  |  |  |
| All patients | -3.49 | 2.84 vs 6 data points | -1.70 | 3.31 vs 6 data points |
| Naïve | -1.68 | 1.47 vs 3 data points | 0.04 | 2.16 vs 3 data points |
| Experienced | -1.00 | 1.52 vs 2 data points | 0.14 | 1.84 vs 2 data points |
| **Progression-free survival** |  |  |  |  |
| All patients | -4.49 | 4.78 vs 6 data point | -2.95 | 4.77 vs 6 data point |
| Naïve | -1.72 | 3.15 vs 3 data point | -0.64 | 2.77 vs 3 data point |
| Experienced | -0.77 | 2.63 vs 3 data point | -0.68 | 2.74 vs 3 data point |
| **Serious AEs** |  |  |  |  |
| All patients | 87.02 | 20.05 vs 12 data points | 83.14 | 13.13 vs 12 data points |
| Naïve | 46.78 | 11.49 vs 6 data points | 44.42 | 7.58 vs 6 data points |
| Experienced | 37.16 | 4.58 vs 6 data points | 37.87 | 4.82 vs 6 data points |
| AE = adverse events, DIC = deviance information criteria. | | | | |

**B. Model diagnosis statistics: Network meta-analyses**

| **Outcome** | **Fixed Effects** | | **Random Effects** | |
| --- | --- | --- | --- | --- |
|  | **DIC** | **Residual deviance** | **DIC** | **Residual deviance** |
| **Overall survival** |  |  |  |  |
| All patients | 6.29 | 10.97 vs 9 data points | 7.62 | 9.97 vs 9 data points |
| Naïve | 6.94 | 8.44 vs 6 data points | 6.71 | 6.34 vs 6 data points |
| Experienced | 2.82 | 3.00 vs 3 data points | 2.79 | 2.99 vs 3 data points |
| **Progression-free survival** |  |  |  |  |
| All patients | -0.85 | 8.51 vs 10 data point | 1.08 | 8.79 vs 10 data point |
| Naïve | -0.58 | 5.19 vs 6 data point | 1.03 | 5.53 vs 6 data point |
| Experienced | 3.41 | 3.99 vs 4 data point | 3.35 | 3.96 vs 4 data point |
| **Serious AEs** |  |  |  |  |
| All patients | 110.9 | 20.56 vs 16 data points | 109.4 | 16.62 vs 16 data points |
| Naïve | 73.24 | 14.41 vs 10 data points | 71.34 | 11.17 vs 10 data points |
| Experienced | 40.68 | 6.08 vs 6 data points | 40.61 | 6.06 vs 6 data points |
| AE = adverse events, DIC = deviance information criteria. | | | | |

**C: Comparison of the consistency and inconsistency models***

*For networks with closed loops (overall survival, serious adverse events)

***Overall survival:***

***Serious adverse events:***

# Appendix 6. Treatment-related death

| **Author, yr, page no. (study name; NCT no.)*** | **Mutation** | **Could participants switch groups?** | | **Treatments  (no. randomized)** | **No. of events /no. who received treatment** | **Cause of death** | **Duration of follow-up, median, mo** |
| --- | --- | --- | --- | --- | --- | --- | --- |
| **Treatment naïve** | | | | | | | |
| Zhou 2019, p. 437 (ALESIA; NCT02838420)^13^ | ALK | No | CRIZ 250 mg BID (62) ALE 600 mg BID (125) | | 2/62 0/125 | Both deaths: interstitial lung disease | 15.0 (IQR 12.5–17.3) 16.2 (IQR 13.7–17.6) |
| Camidge 2018, p. 1 (ALTA-1L; NCT02737501)^10^ | ALK | Yes | CRIZ 250 mg BID (138) BRIG 180 mg QD (137) | | 0/138 0/137 | NA | 9.3 mo (range 0 – 20.9) 11.0 mo (range 0 – 20.0) |
| Peters 2017, p. 829 (ALEX; NCT02075840)^7,15^ | ALK | No | CRIZ 250 mg BID (151)  ALE 600 mg BID (152) | | 2/151  0/152 | NR | 10.7 (range 0-27) 17.9 (range 0-29) |
| Wu 2018, p. (PROFILE 1029; NCT01639001)^12,16^ | ALK | Yes | CHEMO (103) CRIZ 250 mg BID (104) | | 0/101 2/104 | NR | Up to 33 mo |
| Soria 2017, p. 917^9,17^ (ASCEND-4; NCT01828099) | ALK | NR | CHEMO (187) CER 750 mg QD (189) | | 0/175 0/189 | NA | 33 mo |
| Solomon 2014, p. 2167^2^ (PROFILE 1014; NCT01154140) | ALK | Yes | CHEMO (171) CRIZ 250 mg BID (172) | | 0/169 1/171‡ | Pneumonitis | 16.7 mo 17.4 mo |
| **Treatment experienced** | | | | | | | |
| Hida 2017, p. 29  (J-ALEX; JAPICcti-132316)^5^ | ALK | Not during study period | | CRIZ 205 mg BID (104) ALE 300 mg BID (103) | 0/104 0/103 | NA | NR |
| Shaw 2017, p. 874 (ASCEND-5, NCT01828112)^8,14^ | ALK | Yes | | CHEMO (116) CER 750 mg QD (115) | 0/113 0/115 | NA | 16.4 (IQR 11.4-21.4) 16.6 (IQR 11.6-21.4) |
| Hida 2016, p. 1642 (JP28927; JapicCTI-132186)^4^ | ALK | NR | | ALE 300 mg BID† (35) | 0/35 | NA | 13.1 (range 1.1 to 15.0) |
| Zhao 2015, p. 616^3^ | ALK | NR | | CHEMO (14) CRIZ 250 mg BID (14) | 0/14 0/14 | NA | 12 mo |
| Shaw 2013, p. 2385  (PROFILE 1007; NCT00932893)^1,18^ | ALK | No | | CHEMO (174) CRIZ 250 mg BID (173) | 1/171 3/172 | CHEMO: sepsis (1); CRIZ: arrhythmia (1), interstitial lung disease or pneumonitis (2) | 12.1 12.2 |
| Note: ALE = alectinib, BID = twice daily, BRIG = brigatinib, CER = ceritinib, CHEMO = chemotherapy, CRIZ = crizotinib, IQR = interquartile range, NA = not applicable, NR = not reported, QD = once daily. *Author, year for the primary publication of study results. Where updated data were provided in a subsequent companion, updated data were used in this analysis. †30-day crossover: 10 days each of 20/40 mg capsules (fasting) <-> 150 mg capsules (fasting), followed by 10-day extension during which all participants received 150 mg ALE (non-fasting). ‡The participant who died was initially randomized to chemotherapy and switched to crizotinib. | | | | | | | |

**Treatment-related deaths: Relative risks and odds ratios (Bayesian meta-analysis)**

| **TR deaths** | **Relative risk (95% Credible Interval)** | **Odds ratio (95% Credible Interval)** |
| --- | --- | --- |
| All participants | 2.57 (0.76, 11.25) | 2.59 (0.76, 11.37) |
| Experienced | 2.22 (0.40, 19.24) | 2.23 (0.39, 19.66) |
| Naïve | 2.57 (0.76, 11.25) | 2.59 (0.76, 11.37) |

# Appendix 7. Overall survival

**A) Evidence summary**

| **Author, yr, page  (study name; NCT no)*** | **Mutation** | **Could participants switch groups?** | **Treatments  (no. randomized)** | **Survival at 12 mo, % (95% CI)** | **Overall survival, HR (95% CI)** |
| --- | --- | --- | --- | --- | --- |
| **Treatment naive** | | | | | |
| Zhou 2019, p. 437 (ALESIA; NCT02838420)^13^ | ALK | No | CRIZ 250 mg BID (62)  ALE 600 mg BID (125) | NR | REF  0.28 (0.12–0.68)‡ |
| Camidge 2018, p. 1 (ALTA-1L; NCT02737501)^10^ | ALK | Yes | CRIZ 250 mg BID (138) BRIG 180 mg QD (137) | 86 (77, 91) 85 (76, 91) | REF  0.98 (0.50, 1.93) |
| Peters 2017, p. 829 (ALEX; NCT02075840)^7,15,19^ | ALK | No | CRIZ 250 mg BID (151)  ALE 600 mg BID (152) | 82.5 (76.1, 88.9) 84.3 (78.4, 90.2) | REF 0.76 (0.50, 1.15)‡ |
| Wu 2018, p.1539 (PROFILE 1029, NCT01639001)^12,16^ | ALK | NR | CHEMO (103) CRIZ 250 mg BID (104) | 79.5 (70.0, 86.2) 79.3 (70.0, 86.0) | REF 0.897 (0.556, 1.445) |
| Soria 2017, p. 917^9,17^ (ASCEND-4; NCT01828099) | ALK | Yes | CHEMO (187) CER 750 mg QD (189) | 78.7 (NR) 84.3 (NR) | REF 0.73 (0.50, 1.08) |
| Solomon 2014, p. 2167^2,20^ (PROFILE 1014; NCT01154140) | ALK | Yes | CHEMO (171) CRIZ 250 mg BID (172) | 78.4 (71.3, 83.9) 83.5 (77.0, 88.3) | REF 0.760 (0.548, 1.053) |
| **Treatment experienced** | | | | | |
| Novello 2018, p. 1409 (ALUR; NCT02604342)^11^ | ALK | YES | CHEMO (35) ALE 600 mg BID (72) | NR | REF 0.89 (0.35, 2.24)‡ |
| Shaw 2017, p. 874 (ASCEND-5, NCT01828112)^8,14^ | ALK | Yes | CHEMO (116) CER 750 mg QD (115) | NR | REF 1.02 (0.68, 1.54)‡ |
| Shaw 2013, p. 2385  (PROFILE 1007; NCT00932893)^1,18^ | ALK | No | CHEMO (174)† CRIZ 250 mg BID (173) | 66.7 (77.4, 88.5) 70.4 (62.9, 76.7) | REF 0.854 (0.661. 1.104)‡ |
| Note: ALE = alectinib, BID = twice daily, CER = ceritinib, CHEMO = chemotherapy, CI = confidence interval, CRIZ = crizotinib, HR = hazard ratio, NA = not applicable, NR = not reported, QD = once daily, REF = reference group. *Author, year for the primary publication of study results. Where updated data were provided in a subsequent companion report, updated data were used in this analysis. †Participants in the chemotherapy group with disease progression could later enrol in PROFILE 1005. ‡Immature. | | | | | |

**B) Meta-analysis of each treatment pair included in the evidence network (direct evidence); All participants: Overall survival**

| **Comparison** | **No. of studies** | ***I*^2^ (%)** | **Hazard ratio (95%CrI)*** |
| --- | --- | --- | --- |
| CRIZ 250 BID v. CHEMO | 3 | 0 | 0.83 (0.69, 1.00) |
| CERT 750 QD v. CHEMO | 2 | 27 | 0.85 (0.64, 1.13) |
| CRIZ 250 BID v. ALECT 600 BID | 2 | 76 | 0.63 (0.43, 0.91) |
| ALECT 600 BID v. CHEMO | 1 | NA | 0.89 (0.35, 2.25) |
| CRIZ 250 BID v. BRIG 180 QD | 1 | NA | 0.98 (0.50, 1.93) |
| Note: ALE = alectinib, BID = twice daily, CER = ceritinib, CHEMO = chemotherapy, CrI = credible interval, CRIZ = crizotinib, NA = not applicable, QD = once daily. *Fixed-effects model | | | |

**C) Network meta-analysis of ALK inhibitors among (i) treatment-naïve participants and (ii) treatment-experienced participants — Overall survival**

**(i) Treatment-naïve participants**

***Evidence network***

**
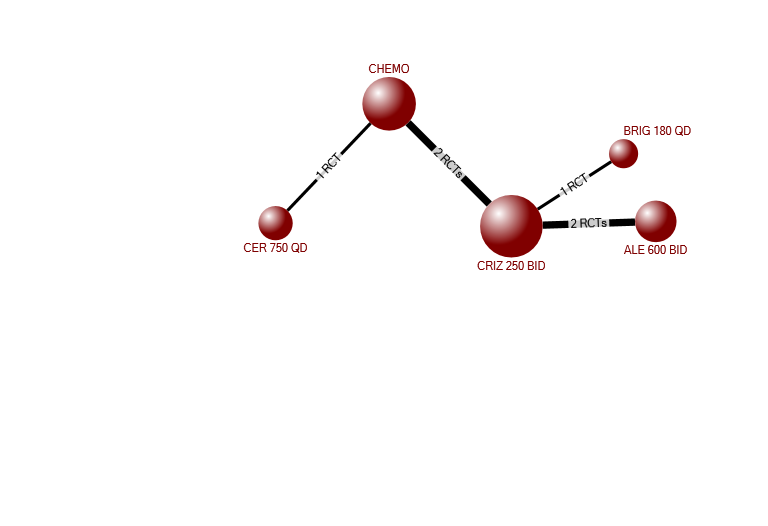
**

***Overall survival – Network meta-analysis of ALK inhibitors, treatment-naïve participants***

|  | **Hazard ratio (95% credible interval)*** | | | |
| --- | --- | --- | --- | --- |
|  | **CHEMO** | **CRIZ 250 BID** | **CER 750 QD** | **ALE 600 BID** |
| **CRIZ 250 BID** | 0.80 (0.61, 1.05) | — |  |  |
| **CER 750 QD** | 0.73 (0.50, 1.08) | 0.91 (0.57, 1.46) | — |  |
| **ALE 600 BID** | **0.51 (0.32, 0.80)** | **0.63 (0.43, 0.92)** | 0.69 (0.38, 1.26) | — |
| **BRIG 180 QD** | 0.79 (0.38, 1.61) | 0.98 (0.50, 1.91) | 1.07 (0.47, 2.41) | 1.55 (0.72, 3.34) |
| Note: ALE = alectinib, BID = twice daily, BRIG = brigatinib, CER = ceritinib, CHEMO = chemotherapy, CRIZ = crizotinib, QD = once daily. *Fixed-effects model. Significant changes are indicated by use of bold and colour (green indicates that the row treatment is significantly better than the column treatment, while red indicates that the row treatment is significantly worse than the column treatment). White indicates no significant difference between treatments. | | | | |

**(ii) Treatment-experienced participants**

***Evidence network***

**
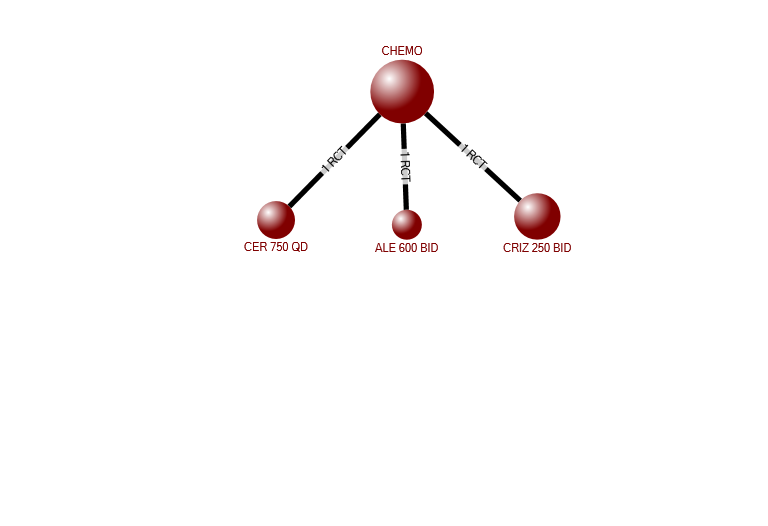
**

***Overall survival – Network meta-analysis of ALK inhibitors, treatment-experienced participants***

|  | **Hazard ratio (95% credible interval)*** | | |
| --- | --- | --- | --- |
|  | **CHEMO** | **CRIZ 250 BID** | **CER 750 QD** |
| **CRIZ 250 BID** | 0.85 (0.66, 1.10) | — |  |
| **CER 750 QD** | 1.02 (0.68, 1.54) | 1.19 (0.74, 1.94) | — |
| **ALE 600 BID** | 0.89 (0.35, 2.24) | 1.04 (0.40, 2.74) | 0.87 (0.31, 2.39) |
| Note: ALE = alectinib, BID = twice daily, CER = ceritinib, CHEMO = chemotherapy, CRIZ = crizotinib, QD = once daily. *Fixed-effects model. Significant changes are indicated by use of bold and colour (green indicates that the row treatment is significantly better than the column treatment, while red indicates that the row treatment is significantly worse than the column treatment). White indicates no significant difference between treatments. | | | |

# Appendix 8. Progression-free survival

**A) Evidence summary**

| **Author, yr, page  (study name)*** | **Mutation** | **Treatments  (no. randomized)** | **PFS at 12 months, % of participants (95% CI)** | **Hazard ratio (95% CI)** |
| --- | --- | --- | --- | --- |
| **Treatment naïve** | | | | |
| Zhou 2019, p. 437 (ALESIA; NCT02838420)^13^ | ALK | CRIZ 250 mg BID (62)  ALE 600 mg BID (125) | NR | REF 0.22 (0.13, 0.38) |
| Camidge 2018, p. 1 (ALTA-1L; NCT02737501)^10^ | ALK | CRIZ 250 mg BID (138) BRIG 180 mg QD (137) | 43 (32, 53) 67 (56, 75) | REF 0.49 (0.33, 0.74) |
| Peters 2017, p. 829 (ALEX)^7,15,19^ | ALK | CRIZ 250 mg BID (151)  ALE 600 mg BID (152) | 48.7 (40.4, 56.9) 68.4 (61.0, 75.9) | REF 0.43 (0.32, 0.58) |
| Wu 2018, p.1539 (PROFILE 1029, NCT01639001)^12,16^ | ALK | CHEMO (103) CRIZ 250 mg BID (104) | NR | REF 0.402 (0.286, 0.565) |
| Soria 2017, p. 917 (ASCEND-4) ^9,17^ | ALK | CHEMO (187) CER 750 mg QD (189) | 39.4 (NR) 58.9 (NR) | REF  0.55 (0.42, 0.73) |
| Solomon 2014, p. 2167^2,20^ (PROFILE 1014) | ALK | CHEMO (171) CRIZ 250 mg BID (172) | NR | REF 0.45 (0.35, 0.60) |
| **Treatment experienced** | | | | |
| Novello 2018, p. 1409 (ALUR; NCT02604342)^11^ | ALK | CHEMO (35) ALE 600 mg BID (72) | NR | REF  0.15 (0.08–0.29) |
| Hida 2017, p. 29 (J-ALEX)^5^ | ALK | CRIZ 205 mg BID (104) ALE 300 mg BID (103) | NR | REF 0.34 (0.17, 0.71) |
| Kim 2017 (ALTA)^6,21^ | ALK | BRI 90 mg QD (112) BRI 180 mg QD (110) | 39.5 (NR) 54.0 (NR) | NR |
| Shaw 2017, p. 874 (ASCEND-5)^8,14^ | ALK | CHEMO (116) CER 750 mg QD (115) | 5.9 (NR) 18.2 (NR) | REF 0.49 (0.36, 0.67) |
| Zhao 2015, p. 616^3^ | ALK | CHEMO (14) CRIZ 250 mg BID (14) | NR | NR |
| Shaw 2013, p. 2385  (PROFILE 1007)^1,18^ | ALK | CHEMO (174) CRIZ 250 mg BID (173) | 11.2 (NR) 25.5 (NR) | REF 0.49 (0.37, 0.64) |
| Note: ALE = alectinib, BID = twice daily, CER = ceritinib, CHEMO = chemotherapy, CI = confidence interval, CRIZ = crizotinib, NR = not reported, PFS = progression-free survival, QD = once daily, REF = reference group. *Author, year for the primary publication of study results. Where updated data were provided in a subsequent companion report, updated data were used in this analysis. | | | | |

**B) Meta-analysis of each treatment pair included in the evidence network (direct evidence); All participants, Progression-free survival**

| **Comparison** | **No. of studies** | ***I*^2^ (%)** | **Hazard ratio (95%CrI)*** |
| --- | --- | --- | --- |
| CRIZ v. CHEMO | 3 | 0 | 0.45 (0.38, 0.53) |
| CERT v. CHEMO | 2 | 0 | 0.52 (0.42, 0.64) |
| CRIZ v. ALECT (600 BID) | 2 | 0 | 0.46 (0.35, 0.60) |
| CRIZ v. ALECT (300 BID) | 1 | NA | 0.34 (0.17, 0.69) |
| CRIZ v. BRIG | 1 | NA | 0.49 (0.33, 0.73) |
| ALECT v. CHEMO | 1 | NA | 0.32 (0.17, 0.60) |
| Note: ALE = alectinib, BID = twice daily, CER = ceritinib, CHEMO = chemotherapy, CI = credible interval, CRIZ = crizotinib, NA = not applicable, QD = once daily. *Fixed-effects model | | | |

**C) Network meta-analysis of ALK inhibitors among (i) treatment-naïve participants and (ii) treatment-experienced participants: progression-free survival**

**(i) Treatment-naïve participants**

***Evidence network***


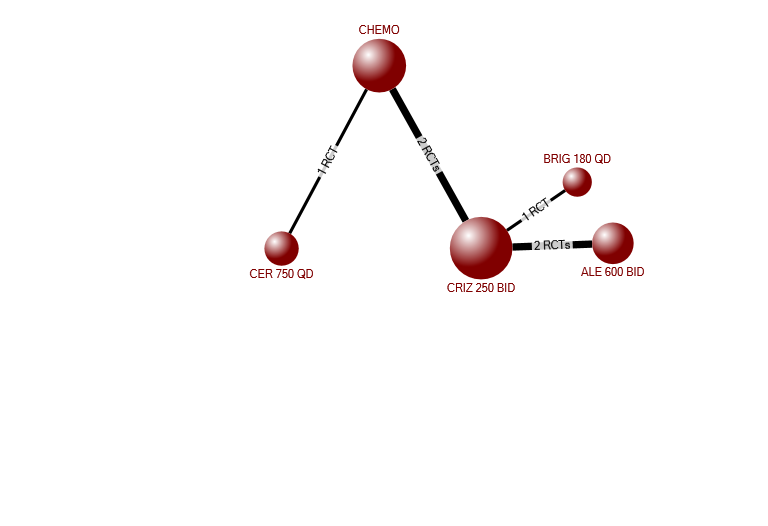


***Progression-free survival – Network meta-analysis of ALK inhibitors, treatment-naïve participants***

|  | **Hazard ratio (95% credible interval)*** | | | |
| --- | --- | --- | --- | --- |
|  | **CHEMO** | **CRIZ 250 BID** | **CER 750 QD** | **ALE 600 BID** |
| **CRIZ 250 BID** | 0.43 (0.35, 0.53) | — |  |  |
| **CER 750 QD** | 0.55 (0.42, 0.73) | 1.28 (0.90, 1.81) | — |  |
| **ALE 600 BID** | 0.20 (0.14, 0.28) | 0.46 (0.35, 0.60) | 0.36 (0.23, 0.56) | — |
| **BRIG 180 QD** | 0.21 (0.13, 0.33) | 0.49 (0.33, 0.73) | 0.38 (0.23, 0.65) | 1.07 (0.66, 1.75) |
| Note: ALE = alectinib, BID = twice daily, CER = ceritinib, CHEMO = chemotherapy, CRIZ = crizotinib, QD = once daily. *Fixed-effects model. Significant changes are indicated by use of bold and colour (green indicates that the row treatment is significantly better than the column treatment, while red indicates that the row treatment is significantly worse than the column treatment). White indicates no significant difference between treatments. | | | | |

**(ii) Treatment-experienced participants**

***Evidence network***


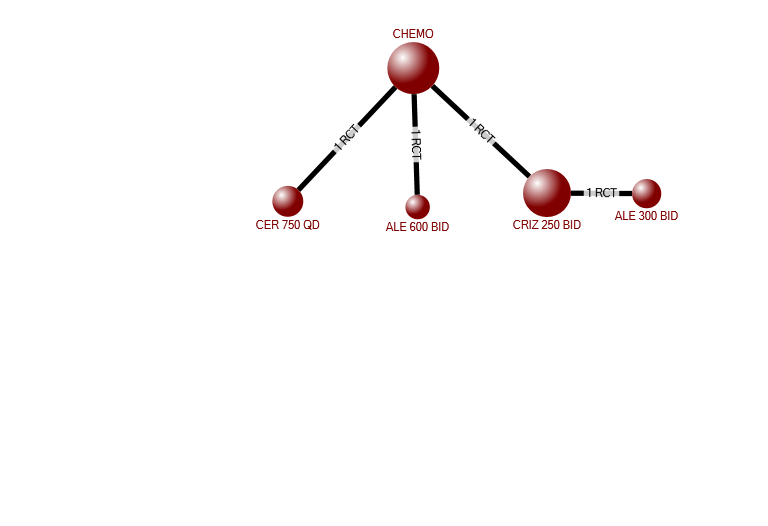


***Progression-free survival – Network meta-analysis of ALK inhibitors, treatment-experienced participants***

|  | **Hazard ratio (95% credible interval)*** | | | |
| --- | --- | --- | --- | --- |
|  | **CHEMO** | **CRIZ 250 BID** | **CER 750 QD** | **ALE 600 BID** |
| **CRIZ 250 BID** | 0.49 (0.37, 0.64) | — |  |  |
| **CER 750 QD** | 0.49 (0.36, 0.67) | 1.00 (0.66, 1.51) | — |  |
| **ALE 600 BID** | 0.17 (0.08, 0.36) | 0.34 (0.17, 0.69) | 0.34 (0.15, 0.77) | — |
| **ALE 300 BID** | 0.32 (0.17, 0.59) | 0.65 (0.33, 1.28) | 0.65 (0.32,1.30) | 1.92 (0.72,5.14) |
| Note: ALE = alectinib, BID = twice daily, CER = ceritinib, CRIZ = crizotinib, QD = once daily. *Fixed-effects model. Significant changes are indicated by use of bold and colour (green indicates that the row treatment is significantly better than the column treatment, while red indicates that the row treatment is significantly worse than the column treatment). White indicates no significant difference between treatments. | | | | |

# Appendix 9. Serious adverse events

**A) Evidence summary**

| **Author, yr, page (study name)*** | **Mutation** | **Could participants switch?** | **Treatments  (no. randomized)** | **No. of events /no. in analysis** |
| --- | --- | --- | --- | --- |
| **Treatment naïve** | | |  | |
| Zhou 2019, p. 437 (ALESIA; NCT02838420)^13^ | ALK | No | CRIZ 250 mg BID (62)  ALE 600 mg BID (125) | 16/62 19/125 |
| Peters 2017, p. 829 (ALEX; NCT02075840)^7,15^ | ALK | Yes | CRIZ 250 mg BID (151)  ALE 600 mg BID (152) | 45/151 44/152 |
| Wu 2018, p. (PROFILE 1029; NCT01639001)^12,16^ | ALK | NR | CHEMO (103) CRIZ 250 mg BID (104) | 13/101 34/104 |
| Solomon 2014, p. 2167^2^ (PROFILE 1014; NCT01154140) | ALK | Yes | CHEMO (171) CRIZ 250 mg BID (172) | 49/169 71/171 |
| Soria 2017, p. 917^9,17^ (ASCEND-4; NCT01828099) | ALK | Yes | CHEMO (187) CER 750 mg QD (189) | 62/175 70/189 |
| **Treatment experienced** | | |  | |
| Novello 2018, p. 1409 (ALUR; NCT02604342)^11^ | ALK | Yes | CHEMO (35) ALE 600 mg BID (72) | 5/34 13/70 |
| Kim 2017 (ALTA, NCT02094573)^6,21^ | ALK | Yes | BRI 90 mg QD (112) BRI 180 mg QD (110) | 41/109 44/110 |
| Shaw 2017, p. 874 (ASCEND-5, NCT01828112)^8,14^ | ALK | Yes | CHEMO (116) CER 750 mg QD (115) | 36/113 49/115 |
| Shaw 2013, p. 2385  (PROFILE 1007; NCT00932893)^1,18^ | ALK | No | CHEMO (174) CRIZ 250 mg BID (173) | 42/171 80/172 |
| Note: ALE = alectinib, BID = twice daily, BRI = brigatinib, CER = ceritinib, CHEMO = chemotherapy, CRIZ = crizotinib, QD = once daily.  *In each trial, SAE data were extracted from the ClinicalTrials.gov records, defined as “an adverse event that results in death, is life threatening, requires inpatient hospitalization or extends a current hospital stay, results in ongoing or significant incapacity or interferes substantially with normal life functions, or causes a congenital anomaly or birth defect” (ClinicalTrials.gov) †Author, year for the primary publication of study results. Where updated data were provided in a subsequent companion report, updated data were used in this analysis. | | | | |

**B) Serious adverse events deaths: Relative risks and odds ratios (Bayesian meta-analysis)**

| **TR deaths** | **Relative risk (95% Credible Interval)** | **Odds ratio (95% Credible Interval)** |
| --- | --- | --- |
| All pts | 1.44 (1.23, 1.69) | 1.67 (1.34, 2.08) |
| Experienced | 1.60 (1.20, 2.17) | 1.75 (1.24, 2.46) |
| Naïve | 1.28 (1.05, 1.57) | 1.42 (1.07, 1.89) |

**C) Meta-analysis of each treatment pair included in the evidence network (direct evidence); All participants, Serious adverse events**

| **Comparison** | **No. of studies** | ***I*^2^ (%)** | **Odds ratio (95%CrI)*** |
| --- | --- | --- | --- |
| CRIZ 250 BID v. CHEMO | 3 | 69 | 2.11 (1.57, 2.85) |
| CERT 750 QD v. CHEMO | 2 | 18 | 1.25 (0.90, 1.75) |
| CRIZ 250 BID v. ALECT 600 BID | 2 | 50 | 0.80 (0.52, 1.21) |
| ALECT 600 BID v. CHEMO | 1 | NA | 1.32 (0.43, 4.07) |
| Note: ALE = alectinib, BID = twice daily, CER = ceritinib, CHEMO = chemotherapy, CrI = credible interval, CRIZ = crizotinib, NA = not applicable, QD = once daily. *Fixed-effects model | | | |

**D) Network meta-analysis for serious adverse events among all participants**

***Evidence network***

***
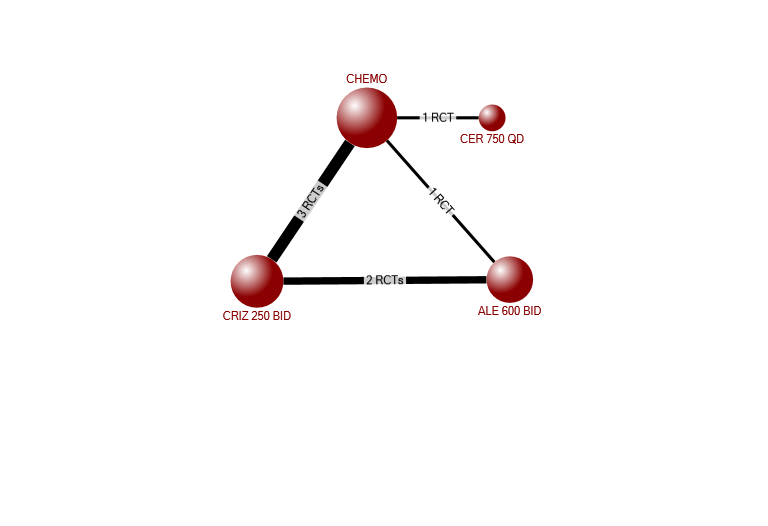
***

***Serious adverse events — Network meta-analysis* *of ALK inhibitors among all participants***

|  | **Relative risk (95% credible interval); Odds ratio (95% credible interval)** | | | |
| --- | --- | --- | --- | --- |
|  | **CHEMO** | **CRIZ 250 BID** | **CER 750 QD** | **ALE 600 BID** |
| **CRIZ 250 BID** | **1.66 (1.36, 2.02); 2.08 (1.56, 2.79);** | — |  |  |
| **CER 750 QD** | 1.18 (0.92, 1.49) 1.25 (0.90-1.74) | **0.72 (0.52, 0.95);  0.60 (0.39, 0.93)** | — |  |
| **ALE 600 BID** | **1.40 (1.00, 1.92);**  **1.60 (1.00, 2.58)** | 0.85 (0.64, 1.09);  0.77 (0.52, 1.15) | 1.19 (0.79, 1.77);  1.29 (0.72, 2.30) | — |
| Note: ALE = alectinib, BID = twice daily, CER = ceritinib, CHEMO = chemotherapy, CRIZ = crizotinib.  *Fixed-effects model. Significant changes are indicated by use of bold and colour (green indicates that the row treatment is significantly better than the column treatment, while red indicates that the row treatment is significantly worse than the column treatment). White indicates no significant difference between treatments. | | | | |

**E) Network meta-analysis of ALK inhibitors among (i) treatment-naïve participants and (ii) treatment-experienced participants: serious adverse events**

**i) Treatment-naïve participants**

***Evidence network***


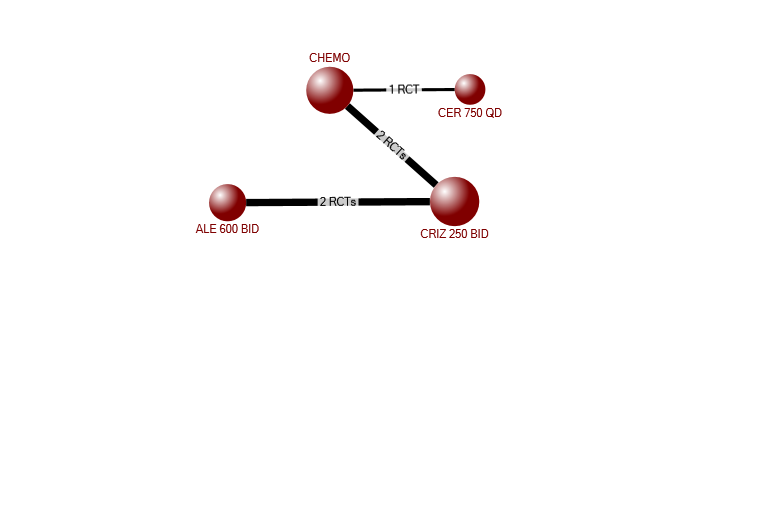


***Serious adverse events — Network meta-analysis* *ALK inhibitors among treatment-naïve participants***

|  | **Relative risk (95% credible interval);**  **Odds ratio (95% credible interval)** | | | |
| --- | --- | --- | --- | --- |
|  | **CHEMO** | **CRIZ 250 BID** | **CER 750 QD** | **ALE 600 BID** |
| **CRIZ 250 BID** | **1.48 (1.14,1.93);  1.77 (1.20, 2.63)** | — |  |  |
| **CER 750 QD** | 1.05 (0.76, 1.43);  1.07 (0.70, 1.65) | 0.71 (0.47, 1.05); 0.60 (0.34, 1.07) | — |  |
| **ALE 600 BID** | 1.28 (0.84, 1.84);  1.41 (0.79, 2.48) | 0.86 (0.63, 1.12);  0.80 (0.52, 1.21) | 1.22 (0.73, 1.97);  1.32 (0.65, 2.67) | — |
| Note: ALE = alectinib, BID = twice daily, CHEMO = chemotherapy, CER = ceritinib, CRIZ = crizotinib.  *Fixed-effects model. Significant changes are indicated by use of bold and colour (green indicates that the row treatment is significantly better than the column treatment, while red indicates that the row treatment is significantly worse than the column treatment). White indicates no significant difference between treatments. | | | | |

**(ii) Treatment-experienced participants**

***Evidence network***

**
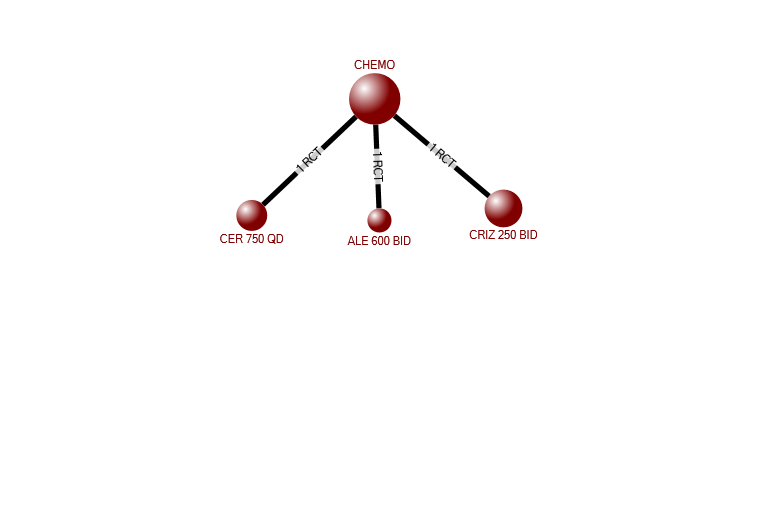
**

***Serious adverse events — Network meta-analysis* *of ALK inhibitors among treatment-experienced participants***

|  | **Relative risk (95% credible interval); Odds ratio (95% credible interval)** | | | |
| --- | --- | --- | --- | --- |
|  | **CHEMO** | **CRIZ 250 BID** | **CER 750 QD** | **ALE 600 BID** |
| **CRIZ 250 BID** | **1.61 (1.17, 2.20); 1.95 (1.23, 3.14)** | — |  |  |
| **CER 750 QD** | 1.41 (0.95, 2.05); 1.60 (0.93, 2.76) | 0.88 (0.33, 2.34);  0.82 (0.40, 1.69) | — |  |
| **ALE 600 BID** | 1.27 (0.53, 2.96);  1.37 (0.45, 4.91) | 0.81 (0.24, 2.58);  0.71 (0.21, 2.69) | 0.90 (0.36, 2.22); 0.86 (0.25, 3.38) | — |
| Note: BID = twice daily, CER = ceritinib, CHEMO = chemotherapy, CRIZ = crizotinib.  *Fixed-effects model. Significant changes are indicated by use of bold and colour (green indicates that the row treatment is significantly better than the column treatment, while red indicates that the row treatment is significantly worse than the column treatment). White indicates no significant difference between treatments. | | | | |


**References**

1. Shaw AT, Kim DW, Nakagawa K, et al. Crizotinib versus chemotherapy in advanced ALK-positive lung cancer. *New England Journal of Medicine.* 2013;368:2385-94.

2. Solomon BJ, Mok T, Kim DW, et al. First-line crizotinib versus chemotherapy in ALK-positive lung cancer. *New England Journal of Medicine.* 2014;371:2167-77.

3. Zhao J, Zhang K, Zhang L, Wang H. Clinical Efficacy of Crizotinib in Advanced ALK Positive Non-small Cell Lung Cancer. *Zhongguo Fei Ai Za Zhi.* 2015;18:616-20.

4. Hida T, Nakagawa K, Seto T, et al. Pharmacologic study (JP28927) of alectinib in Japanese patients with ALK+ non-small-cell lung cancer with or without prior crizotinib therapy. *Cancer Science.* 2016;107:1642-6.

5. Hida T, Nokihara H, Kondo M, et al. Alectinib versus crizotinib in patients with ALK-positive non-small-cell lung cancer (J-ALEX): an open-label, randomised phase 3 trial. *Lancet.* 2017.

6. Kim DW, Tiseo M, Ahn MJ, et al. Brigatinib in patients with crizotinib-refractory anaplastic lymphoma kinase-positive non-small-cell lung cancer: a randomized, multicenter phase II trial. *J Clin Oncol.* 2017;JCO2016715904.

7. Peters S, Camidge DR, Shaw AT, et al. Alectinib versus crizotinib in untreated ALK-positive non-small-cell lung cancer. *The New England journal of medicine.* 2017;377:828-38.

8. Shaw AT, Kim TM, Crino L, et al. Ceritinib versus chemotherapy in patients with ALK-rearranged non-small-cell lung cancer previously given chemotherapy and crizotinib (ASCEND-5): a randomised, controlled, open-label, phase 3 trial. *Lancet Oncology.* 2017.

9. Soria JC, Tan DS, Chiari R, et al. First-line ceritinib versus platinum-based chemotherapy in advanced ALK-rearranged non-small-cell lung cancer (ASCEND-4): a randomised, open-label, phase 3 study. *Lancet.* 2017;389:917-29.

10. Camidge DR, Kim HR, Ahn MJ, et al. Brigatinib versus Crizotinib in ALK-Positive Non-Small-Cell Lung Cancer. *New England Journal of Medicine.* 2018;379:2027-39.

11. Novello S, Mazieres J, Oh IJ, et al. Alectinib versus chemotherapy in crizotinib-pretreated anaplastic lymphoma kinase (ALK)-positive non-small-cell lung cancer: results from the phase III ALUR study. *Ann Oncol.* 2018;29:1409-16.

12. Wu YL, Lu S, Lu Y, et al. Results of PROFILE 1029, a Phase III Comparison of First-Line Crizotinib versus Chemotherapy in East Asian Patients with ALK-Positive Advanced Non-Small Cell Lung Cancer. *J Thorac Oncol.* 2018;13:1539-48.

13. Zhou C, Kim SW, Reungwetwattana T, et al. Alectinib versus crizotinib in untreated Asian patients with anaplastic lymphoma kinase-positive non-small-cell lung cancer (ALESIA): a randomised phase 3 study. *Lancet Respir Med.* 2019;7:437-46.

14. Novartis P, Novartis. LDK378 Versus Chemotherapy in ALK Rearranged (ALK Positive) Patients Previously Treated With Chemotherapy (Platinum Doublet) and Crizotinib.

15. Hoffmann-La R. A Study Comparing Alectinib With Crizotinib in Treatment-Naive Anaplastic Lymphoma Kinase-Positive Advanced Non-Small Cell Lung Cancer Participants.

16. Pfizer. A Study Of Crizotinib Versus Chemotherapy In Previously Untreated ALK Positive East Asian Non-Small Cell Lung Cancer Patients. 2017:NCT01639001.

17. Novartis P, Novartis. LDK378 Versus Chemotherapy in Previously Untreated Patients With ALK Rearranged Non-small Cell Lung Cancer.

18. Pfizer. "An Investigational Drug, PF-02341066 Is Being Studied Versus Standard Of Care In Patients With Advanced Non-Small Cell Lung Cancer With A Specific Gene Profile Involving The Anaplastic Lymphoma Kinase (ALK) Gene".

19. Camidge DR, Dziadziuszko R, Peters S, et al. Updated Efficacy and Safety Data and Impact of the EML4-ALK Fusion Variant on the Efficacy of Alectinib in Untreated ALK-Positive Advanced Non-Small Cell Lung Cancer in the Global Phase III ALEX Study. *J Thorac Oncol.* 2019;14:1233-43.

20. Solomon BJ, Kim DW, Wu YL, et al. Final Overall Survival Analysis From a Study Comparing First-Line Crizotinib Versus Chemotherapy in ALK-Mutation-Positive Non-Small-Cell Lung Cancer. *J Clin Oncol.* 2018;36:2251-8.

21. Ariad P. "A Study to Evaluate the Efficacy of Brigatinib (AP26113) in Participants With Anaplastic Lymphoma Kinase (ALK)-Positive, Non-small Cell Lung Cancer (NSCLC) Previously Treated With Crizotinib".
